# A compensatory RNase E variation increases Iron Piracy and Virulence in multidrug-resistant *Pseudomonas aeruginosa* during Macrophage infection

**DOI:** 10.1371/journal.ppat.1010942

**Published:** 2023-04-07

**Authors:** Mylene Vaillancourt, Anna Clara Milesi Galdino, Sam P. Limsuwannarot, Diana Celedonio, Elizabeth Dimitrova, Matthew Broerman, Catherine Bresee, Yohei Doi, Janet S. Lee, William C. Parks, Peter Jorth

**Affiliations:** 1 Department of Pathology and Laboratory Medicine, Cedars-Sinai Medical Center, Los Angeles, California, United States of America; 2 Women’s Guild Lung Institute, Department of Medicine, Cedars-Sinai Medical Center, Los Angeles, California, United States of America; 3 Acute Lung Injury Center of Excellence, Division of Pulmonary, Allergy, and Critical Care Medicine, Department of Medicine; Vascular Medicine Institute, University of Pittsburgh, Pittsburgh, Pennsylvania, United States of America; 4 Biostatistics Core, Cedars-Sinai Medical Center, Los Angeles, California, United States of America; 5 Division of Infectious Diseases, Department of Medicine, University of Pittsburgh, Pittsburgh, Pennsylvania, United States of America; 6 Department of Biomedical Sciences, Cedars-Sinai Medical Center, Los Angeles, California, United States of America; Reims Champagne-Ardenne University Faculty of Medicine: Universite de Reims Champagne-Ardenne UFR de Medecine, FRANCE

## Abstract

During chronic cystic fibrosis (CF) infections, evolved *Pseudomonas aeruginosa* antibiotic resistance is linked to increased pulmonary exacerbations, decreased lung function, and hospitalizations. However, the virulence mechanisms underlying worse outcomes caused by antibiotic resistant infections are poorly understood. Here, we investigated evolved aztreonam resistant *P*. *aeruginosa* virulence mechanisms. Using a macrophage infection model combined with genomic and transcriptomic analyses, we show that a compensatory mutation in the *rne* gene, encoding RNase E, increased pyoverdine and pyochelin siderophore gene expression, causing macrophage ferroptosis and lysis. We show that iron-bound pyochelin was sufficient to cause macrophage ferroptosis and lysis, however, apo-pyochelin, iron-bound pyoverdine, or apo-pyoverdine were insufficient to kill macrophages. Macrophage killing could be eliminated by treatment with the iron mimetic gallium. RNase E variants were abundant in clinical isolates, and CF sputum gene expression data show that clinical isolates phenocopied RNase E variant functions during macrophage infection. Together these data show how *P*. *aeruginosa* RNase E variants can cause host damage via increased siderophore production and host cell ferroptosis but may also be targets for gallium precision therapy.

## Author summary

In people with cystic fibrosis (CF), *Pseudomonas aeruginosa* becomes antibiotic resistant during decades-long lung infections and resistance is linked to worse outcomes in people with CF. While resistance clearly reduces treatment efficacy, the virulence functions used by resistant bacteria to drive worse disease are poorly understood. Here we investigated virulence mechanisms in antibiotic resistant *P*. *aeruginosa*. We show that resistant bacteria are more effective at killing macrophage immune cells, and this process is driven by a mutation in a bacterial gene encoding RNase E, a key enzyme in bacteria used to degrade messenger RNAs. We demonstrate that the RNase E mutation causes *P*. *aeruginosa* to overproduce iron scavenging siderophore molecules that, in turn, damage macrophage cell membranes. RNase E variants are also detected in *P*. *aeruginosa* infecting people with CF, suggesting that these variants could help this bacterium fight immune cells during human infections. We also show that gallium, an emerging antibacterial, can reduce virulence of RNase E variants, which indicates that gallium could be used as a precision treatment for these dangerous infections. These findings reveal new roles for RNase E variants and siderophores in bacterial pathogenesis.

## Introduction

*Pseudomonas aeruginosa* is a ubiquitous bacterium that thrives in a wide range of environments including water, soils, plants, and animal tissues [1]. In clinical settings, *P*. *aeruginosa* is one of the prevailing pathogens involved in acute nosocomial infections and chronic lung infections in cystic fibrosis (CF) [2]. During acute infection by *P*. *aeruginosa*, innate immune cells are critical for clearance by rapidly decreasing bacterial loads during the initial infection, even before the first administration of antibiotics [3]. In particular, macrophages are the predominant immune cells to phagocytose *P*. *aeruginosa* after bacterial inhalation [4]. More importantly, bacterial clearance by macrophages prevents neutrophil infiltration and subsequent inflammation [4–6]. During lung infection, recruitment of peripheral monocytes/macrophages is essential for bacterial clearance and reduced neutrophilia [5]. In the same line, lower numbers of activated monocytes/macrophages are associated with the presence of *P*. *aeruginosa* infection and disease severity in CF [7]. These observations underscore a crucial role for macrophages in bacterial clearance and homeostasis of the lung.

Bacteria evolve several mechanisms to escape phagocytosis and colonize the lung environment. For instance, *P*. *aeruginosa* use rhamnolipids, a glycolipid biosurfactant, to break through epithelial barriers and escape phagocytosis by immune cells [8]. Virulent strains also use the type III secretion system (T3SS) to induce macrophage lysis [9,10]. Bacteria can use molecules from lysed host cells for their own benefits. For example, *P*. *aeruginosa* secretes siderophores that scavenge iron from host cells and are recaptured by the bacteria for their metabolic activities [11]. *P*. *aeruginosa* is also able to maintain its growth using anaerobic respiration by denitrification in the CF lung and sputum [12]. While these phenotypes undoubtedly benefit *P*. *aeruginosa* during infection, the importance of these different mechanisms can be unclear due to genotypic and phenotypic diversification [13,14]. Such diversification can lead to the overexpression or genotypic loss of numerous virulence systems, metabolic versatility, and antibiotic resistance [15–17]. As a result, despite extensive work in this field, the response of *P*. *aeruginosa* to different environmental triggers and the downstream mechanisms involved in virulence and lung adaptation remain elusive.

During chronic lung infection, pathogens experience selective pressure from intense antibiotic therapies [18,19]. Over the past decades, extensive use of antibiotics has led to increased prevalence of multidrug- and extensively drug-resistant *P*. *aeruginosa* worldwide [20–22]. Although the acquisition of antibiotic resistance may be correlated with decreased virulence and fitness, multiple studies found associations between multidrug-resistant *P*. *aeruginosa* infections and pulmonary exacerbations, decreased lung function, and hospitalizations in people with CF [2,23]. In line with these clinical observations, multidrug-resistant *P*. *aeruginosa* strains can exhibit virulence evolved from either the resistance mutation or various types of second-site compensatory mutations [24–27]. However, mechanisms by which antibiotic resistance leads to hypervirulence are poorly understand.

Our group recently showed that a loss-of-function mutation in the *mexR* gene, which led to aztreonam resistance by overexpressing MexAB-OprM efflux pump, was sufficient to increase bacterial virulence *in vivo* [27]. This strain also displayed increased swarming motility resulting from a second-site mutation impacting the MexEF-OprN efflux pump [27]. This second-site mutation enhanced virulence *in vivo*, independently of *mexR* and MexAB-OprM. These data highlight the unexpected dangers of antibiotic selection for hypervirulent bacteria and the urgent need to find alternative therapies to eradicate these “superbugs”.

In the present study, we sought to investigate the virulence mechanisms of the *P*. *aeruginosa* AzEvC10 mutant, a PAO1 strain that was evolved under cyclic aztreonam exposure [26]. This strain first evolved a mutation in the *nalD* gene, resulting in overexpression of the MexAB-OprM efflux pump and multidrug resistance. This mutant was also hypervirulent *in vivo* compared to wild-type (WT) PAO1. However, it was unclear whether the AzEvC10 strain’s virulence was dependent upon the *nalD* mutation or a second-site compensatory mutation. Furthermore, the virulence mechanisms upregulated in the AzEvC10 strain remained elusive. Here we determine mechanisms of hypervirulence of the AzEVC10 evolved strain and ask whether this virulence is caused directly by its antibiotic resistance mutation, or via a second-site compensatory mutation. We also determine the host cell types directly affected by this strain’s increased virulence, identify a potential treatment to kill these hypervirulent *P*. *aeruginosa* variants, and reveal similarities between this lab-evolved strain and *P*. *aeruginosa* clinical isolates.

## Results

### Aztreonam-evolved *P*. *aeruginosa* replicates more during macrophage infection

**Fig 1:**
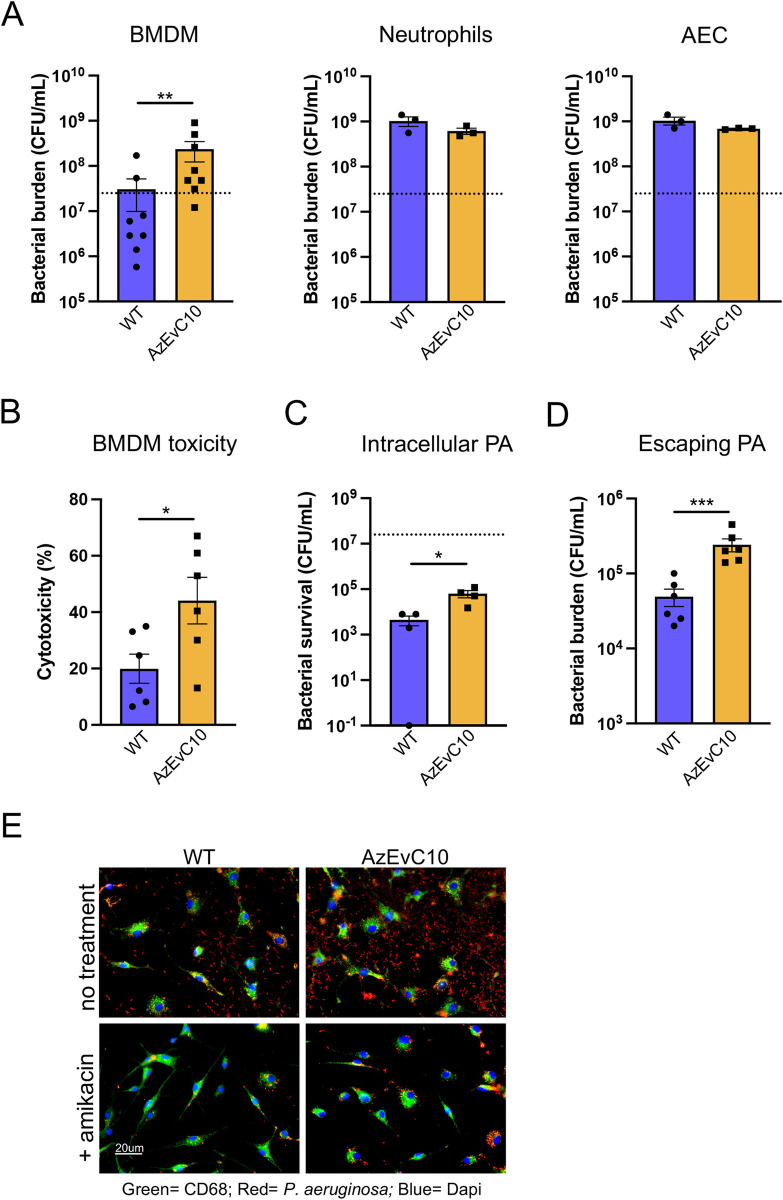
An aztreonam-evolved *P*. *aeruginosa* mutant replicates more during macrophage infection. **A.** BMDM, BM-derived neutrophil, or AEC cells were infected with WT PAO1 or the AzEvC10 mutant for 6 h. Dotted lines represent the initial infection inoculum, 2.5 x 10^7^ CFU/mL. Bacterial burden at 6 hpi was determined by viable CFU plate counts. Analysis was matched for experimental repeat. **B.** BMDM cytotoxicity assessed by LDH assay at 6 hpi by WT or AzEVC10. Analysis was matched for experimental repeat. **C.** Intracellular survival of bacteria in BMDM at 6 hpi, following amikacin treatment at 1 hpi for a total of 5 h to kill extracellular bacteria. Viable intracellular bacteria were determined by CFU plate counts. **D**. Escaping bacteria at 6 hpi: Amikacin was added in medium at 1 hpi and left for 1 h. Then cells were incubated in fresh medium (no amikacin) for another 4 h. Escaping bacteria were determined by CFU plate counts. **E.** Images of BMDM infections at 6 hpi by immunofluorescence showing macrophages in green, *P*. *aeruginosa* in red, and DAPI in blue. n = 3–6 independent replicates for each experiment. *p<0.05, **p<0.01, ***p<0.001, ****p<0.0001. See S5 Table for statistical tests used and exact *p*-values.

To characterize the virulence of the *P*. *aeruginosa* AzEvC10 mutant, we assessed if there was a difference in clearance between the mutant and WT PAO1 by infecting bone marrow-derived macrophages (BMDM), BM-derived neutrophils, or airway epithelial cells (AEC) with 2.5 x 10^7^ CFU/mL of either strain. At 6 h post-infection (hpi) of BMDM, the AzEvC10 mutant had increased in number by about 1 log_10_ whereas the WT parental strain showed no change from the initial inoculum (*p*< 0.01) (Fig 1A). In addition, the AzEvC10 mutant induced higher cytotoxicity of BMDM (44% ± 8.2) compared to the WT strain (20% ± 5.2) (*p*<0.05) (Fig 1B). These differences were specific to BMDM, as there was no difference in the replication of (Fig 1A) or cytotoxicity (S1A Fig) by the two strains in BM-derived neutrophils and AEC. During *in vivo* lung infections, both alveolar macrophages and infiltrated monocytes kill bacteria. Although alveolar macrophages are the first line of defense against pulmonary pathogens and may clear low concentrations of bacteria, recruitment of circulating monocytes/macrophages was shown to be essential to clear higher concentrations of bacteria in the lung [5,28]. For this reason, we chose to perform our infections using BMDM. Their phenotypes were confirmed for alveolar macrophage markers (S1B Fig). We also confirmed the AzEvC10 hypervirulent phenotype during infection of alveolar macrophages (S1C Fig).

We tested if increased proliferation of the AzEvC10 mutant was due to its capacity to use growth medium components as a source of carbon and energy. We found that both strains had net lethality in host cell-free medium but that there was a significant survival difference between the strains. At 6 hpi in cell-free medium, the number of viable WT PAO1 was 3.5 log_10_ less than the initial inoculum, whereas the number of viable AzEvC10 mutants was reduced only 0.8 log_10_ (S1D Fig). These findings demonstrate that the AzEvC10 mutant has acquired a significant survival advantage–nearly 1,000-fold better than WT–in a nutrient restrictive environment.

We also assessed if the survival and subsequent escape of phagocytosed bacteria contributed to the increased bacterial burden of AzEvC10 mutant in macrophage cultures. To test this, we infected BMDM with the mutant and WT and 1 h later added 200 μg/mL amikacin, an antibiotic that does not enter host cells, to kill extracellular bacteria. This concentration of amikacin effectively killed both WT PAO1 and AzEvC10 mutant bacteria (S1E Fig). At 6 hpi (i.e., 5 h post-amikacin), the number of surviving intracellular AzEvC10 mutants was significantly greater (about 1 log_10_) than the number of intracellular viable WT PAO1 (*p*<0.05) (Fig 1C). This could be due to either higher phagocytosis of mutant bacteria by macrophages, increased mutant bacterial proliferation inside host cells, or increased intracellular survival. Regardless, to determine if these intracellular surviving bacteria could escape from BMDM and proliferate extracellularly, we treated BMDM medium with amikacin at 1 hpi for 1 h, then washed the cells and added fresh medium without antibiotic. Compared to WT infected cells, we found that extracellular bacterial counts were about 1 log_10_ increased in AzEvC10 mutant infected cultures at 6 hpi (Fig 1D and 1E), proportional to the difference in viable intracellular bacteria (Fig 1C). These data suggest that both WT PAO1 and AzEvC10 mutant can escape BMDMs and proliferate extracellularly with similar efficiency. Although the specific cause of the increased intracellular bacterial recovery needs to be defined, overall, our studies indicate that AzEvC10 adapts better than WT PAO1 to the host environment and that this growth advantage was enhanced by the presence of macrophages. Below we explore the bacterial and host pathways involved in the mutant’s growth advantage.

### AzEvC10 mutant hypervirulence is caused by a compensatory mutation in RNase E

The aztreonam-evolved AzEvC10 mutant first acquired a loss-of-function point mutation in the *nalD* gene (S2A Fig) in response to the aztreonam selective pressure, which caused overexpression of the MexAB-OprM efflux pump and a multidrug resistance phenotype [26]. Recently, we found that mutation of another *mexAB-oprM* regulator was sufficient to increase *P*. *aeruginosa* virulence [27]. Thus, we hypothesized that the AzEvC10 mutant’s virulence was being driven by its *nalD* mutation. To test this, we complemented a functional wild-type *nalD* gene in the AzEvC10 mutant and infected BMDM to assess if this would reverse the hypervirulence. Complementation of the *nalD* gene was confirmed by mRNA expression quantification and aztreonam MIC (S2B and S2C Fig). Unexpectedly, *nalD* complementation in AzEvC10 did not reverse bacterial growth and cytotoxicity during BMDM infection (S2D and S2E Fig). This was further confirmed when we infected BMDM with mutants in which *mexAB* genes were deleted (S2F–S2I Fig).

**Fig 2:**
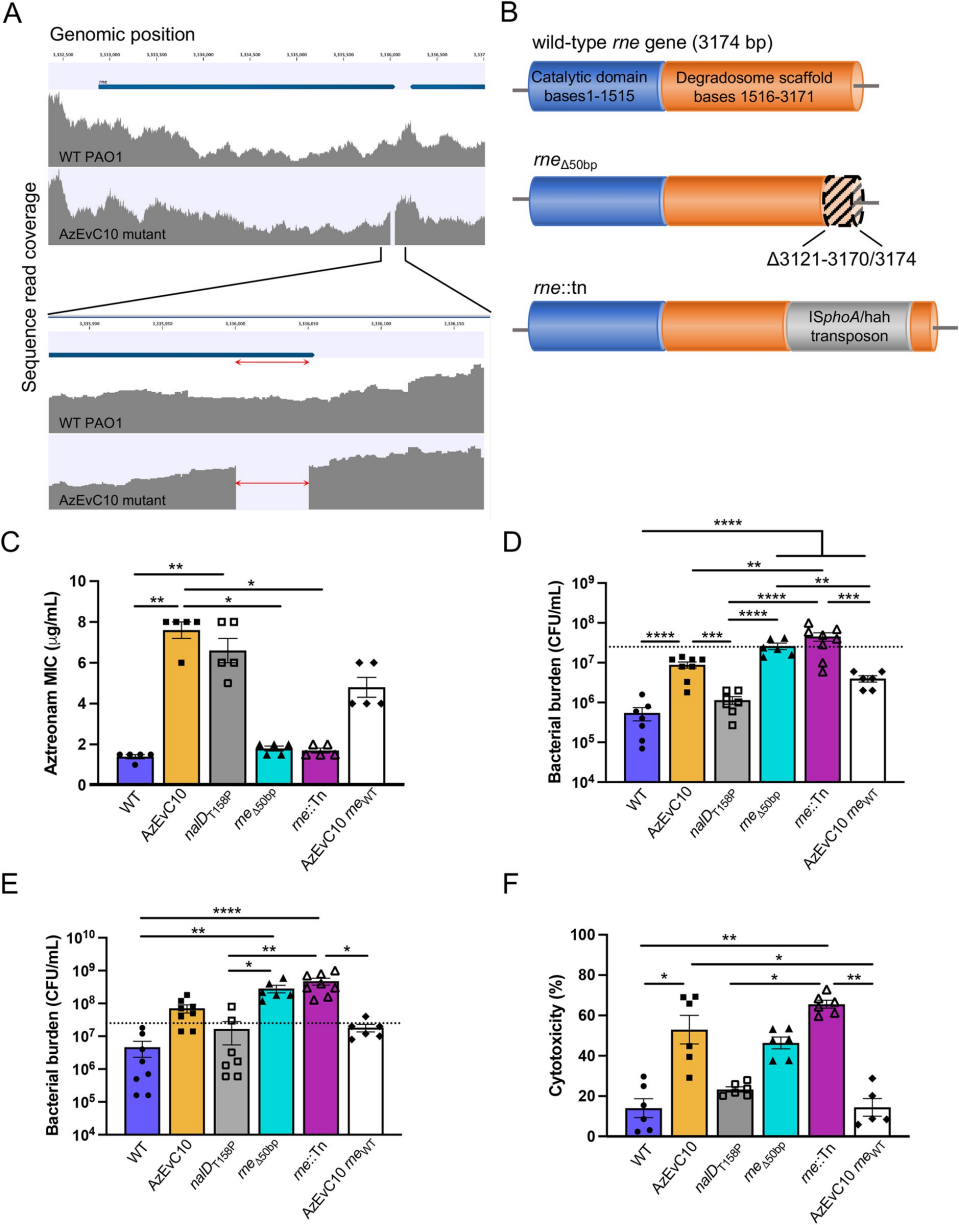
AzEvC10 mutant hypervirulence is caused by an RNase E mutation. **A.** Genome diagram showing coverage of DNA sequencing reads aligning to the *rne* gene. The deletion of bases 3121-3170/3174 nt of the *rne* gene in the AzEvC10 mutant are enlarged in the bottom panel. Genome coverage plots generated from sequencing read alignments to the PAO1 reference genome are indicated in grey. **B.** Schematic representation of the *rne* gene and corresponding RNase E protein domain description. WT PAO1, *nalD*_T158P_, and AzEvC10 *rne*_WT_ mutants carry a wild-type *rne* gene. AzEvC10 and *rne*_Δ50bp_ mutants have a deletion of the bases 3121-3170/3174 nt in the *rne* gene. The *rne*::Tn mutant carries an IS*phoA*/hah transposon inserted at the position 2945 nt of the gene. **C.** Aztreonam MIC was assessed by Etest for the indicated bacteria. **D-E.** BMDM cells were infected with WT PAO1 or different *nalD* and *rne* mutants at MOI:100 for 3 h (**D**) and 6 h (**E**). Bacterial burden was determined by viable CFU plate counts. Dotted lines represent the initial infection inoculum, 2.5 x 10^7^ CFU/mL. **F.** BMDM cytotoxicity was assessed by LDH assay at 6 hpi with indicated bacteria. n = 5–8 independent replicates for each experiment *p<0.05, **p<0.01, ***p<0.001, ****p<0.0001. See S5 Table for statistical tests used and exact *p*-values.

To explore if the AzEvC10 mutant’s hypervirulence could be explained by a second-site compensatory mutation, we used whole genome sequencing and found that the strain evolved a 50-bp deletion (bases 3121-3170/3174) in the *rne* gene (Fig 2A and 2B) encoding RNase E, an endonuclease involved in RNA turnover. Because compensatory mutations can restore fitness and/or virulence, and due to the nature of RNase E activity, we hypothesized that the *rne* variation was involved in the virulence and growth of the AzEvC10 mutant during macrophage infection. To begin testing the virulence consequences of the *rne* mutation, we created single *nalD*_T158P_ and *rne*_Δ50bp_ mutants in a wild-type *P*. *aeruginosa* background and used an *rne* mutant containing a transposon inserted at the position 2945 of the gene (*rne*::Tn) [29]. We also reversed the *rne* mutation directly in the AzEvC10 chromosome at its native position (AzEvC10 *rne*_WT_) as a rescue. As expected, the aztreonam resistance seen in AzEvC10 mutant was caused by the mutation in *nalD* gene (Fig 2C). However, we found that bacterial burden during BMDM infection and BMDM cytotoxicity were increased in AzEvC10, *rne*_Δ50bp_, and *rne*::Tn mutants compared to WT, *nalD*_T158P_, and AzEvC10 *rne*_WT_ strains (Fig 2D–2F). These results confirmed the role of the RNase E variant in the hypervirulent phenotype and growth of the AzEvC10 mutant during macrophage infection.

### Iron acquisition genes are upregulated in the AzEvC10 mutant during BMDM infection

**Fig 3:**
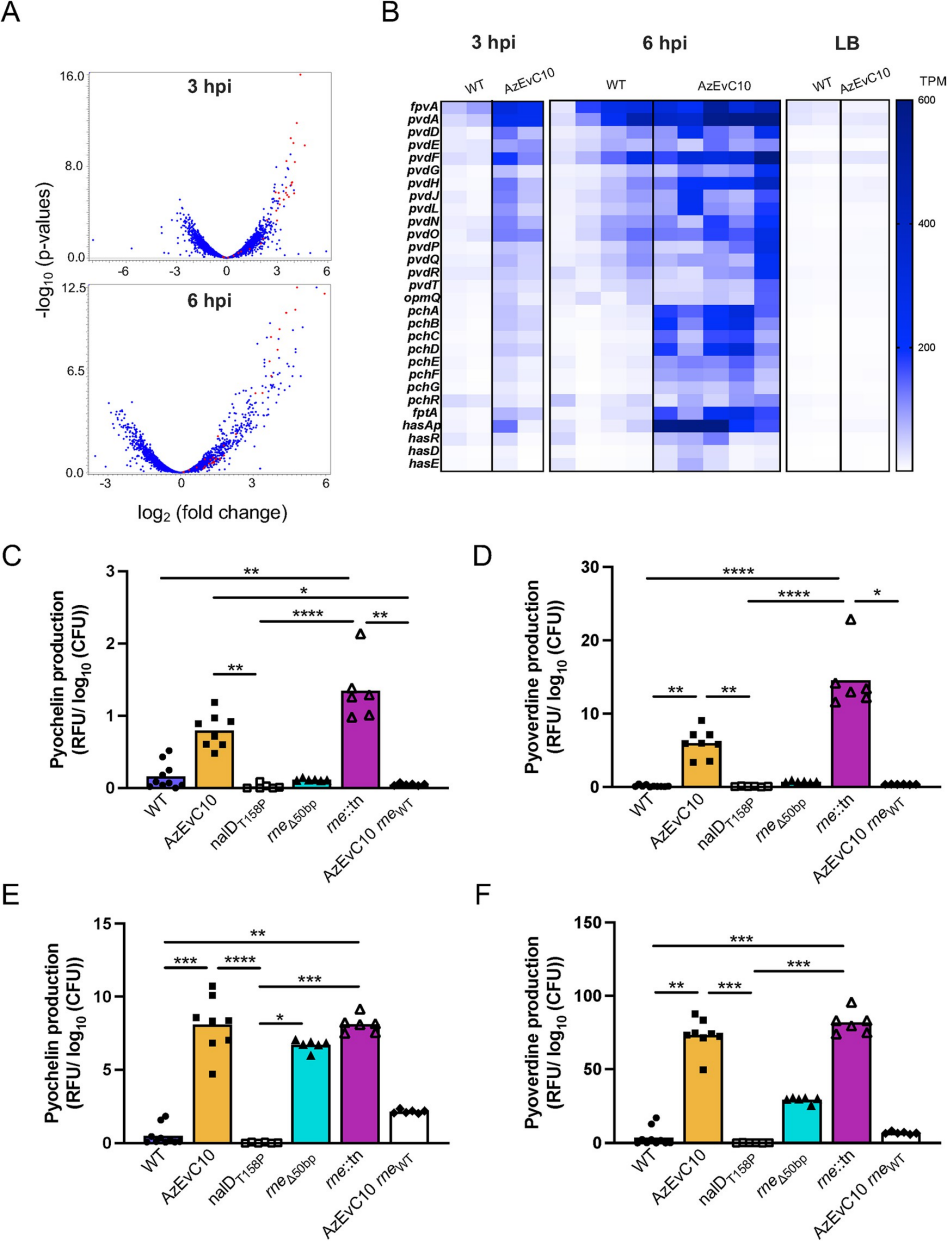
Iron acquisition genes are upregulated in the AzEvC10 mutant during macrophage infection. **A-B.** Genes involved in iron acquisition were upregulated in the AzEvC10 mutant compared to WT PAO1 during BMDM infection at 3 hpi (n = 2) and 6 hpi (n = 4–5), but not when grown in LB broth (n = 2). **A**. Volcano plot highlighting differentially expressed iron acquisition genes in red during BMDM infection. **B**. Heat map indicates expression of indicated genes in individual biological replicates expressed in normalized number of reads (TPM) during WT and AzEvC10 BMDM infections at indicated time points. **C-F.** Pyochelin and pyoverdine production by given strains measured by fluorescence (Ex350/Em430 for pyochelin and Ex400/Em460 for pyoverdine) at 3 hpi **(C-D)** and 6 hpi **(E-F)** and normalized to log_10_(CFU). n = 5–8 independent replicates for each experiment. *p<0.05, **p<0.01, ***p<0.001, ****p<0.0001. See S5 Table for statistical tests used and exact *p*-values.

While the *rne* variation was responsible for the increased virulence of the AzEvC10 mutant, the mechanisms driving the *rne* variant’s increased virulence were unclear. To understand the mechanisms underlying the AzEvC10 mutant phenotype, we infected BMDM and performed RNA sequencing at 3 hpi and 6 hpi. We first compared the AzEvC10 mutant gene expression to WT PAO1 during the BMDM infections at the two timepoints. We found a marked transcriptional upregulation of the two *P*. *aeruginosa* siderophore biogenesis pathways at both 3 hpi and 6 hpi in the AzEvC10 mutant (Fig 3A and 3B). These pathways include genes involved in pyoverdine biosynthesis (*pvdA*, *pvdD*, *pvdE*, *pvdF*, *pvdG*, *pvdH*, *pvdJ*, *pvdL*, *pvdN*,*pvdO*, *pvdP*, *pvdQ*), secretion (*pvdR*, *pvdT*, *opmQ*), and ferric-pyoverdine import (*fpvA*). mRNA from genes encoding the pyochelin biosynthesis enzymes (*pchABCD*, *pchEFG*), ferric-pyochelin import (*fptA*), and their transcriptional regulator (*pchR*) were also upregulated at 3 hpi and even more so at 6 hpi. Transcriptional upregulation of heme acquisition genes (*hasAp*, *hasD*, *hasE and hasR*) was observed but only at 6 hpi. Interestingly, none of these pathways were upregulated at the mRNA level when we grew the strains in LB broth (Fig 3B), suggesting that this mutant’s response was specific to the macrophage infection.

To confirm the effect of the RNase E variation on increased siderophore biogenesis, we measured pyochelin and pyoverdine secreted in the growth medium during BMDM infection. To do this, we measured the fluorescence at Ex350/Em430 for pyochelin and Ex400/Em460 for pyoverdine. The two siderophores had enough fluorescence emission in the macrophage growth medium to be detected (S3A–S3D Fig). A limitation of this technique is that the fluorescence of ferric-pyochelin and ferric-pyoverdine is quenched thus, undetectable (S3–S3D Fig) [30]. Thus, for both siderophores, the fluorescence measurements reflect only a relative quantification of the metal-free forms that was normalized on the log_10_(CFU). At 3 hpi and 6 hpi, both siderophores were barely detectable in WT, *nalD*_T158P_, and AzEvC10 *rne*_WT_ strains (Figs 3C–3F and S3E). In contrast, the AzEvC10 and *rne*::Tn mutants had produced detectable pyochelin and pyoverdine by 3 hpi, and at 6 hpi, the concentrations increased further (Figs 3C–3F and S3E). Surprisingly, the *rne*_Δ50bp_ mutant did not secrete siderophores at 3 hpi, as seen in AzEvC10 and *rne*::Tn mutants (Figs 3C–3D and S3E). However, the mutant did secrete pyochelin and pyoverdine at 6 hpi (Figs 3E–3F and S3E). While the reason for this difference is not clear, it is possible that the dynamics of increased siderophore production vary slightly based on genetic background and the nature of the *rne* variation.

### The AzEvC10 mutant carries specific gene expression signatures during BMDM infection

**Fig 4:**
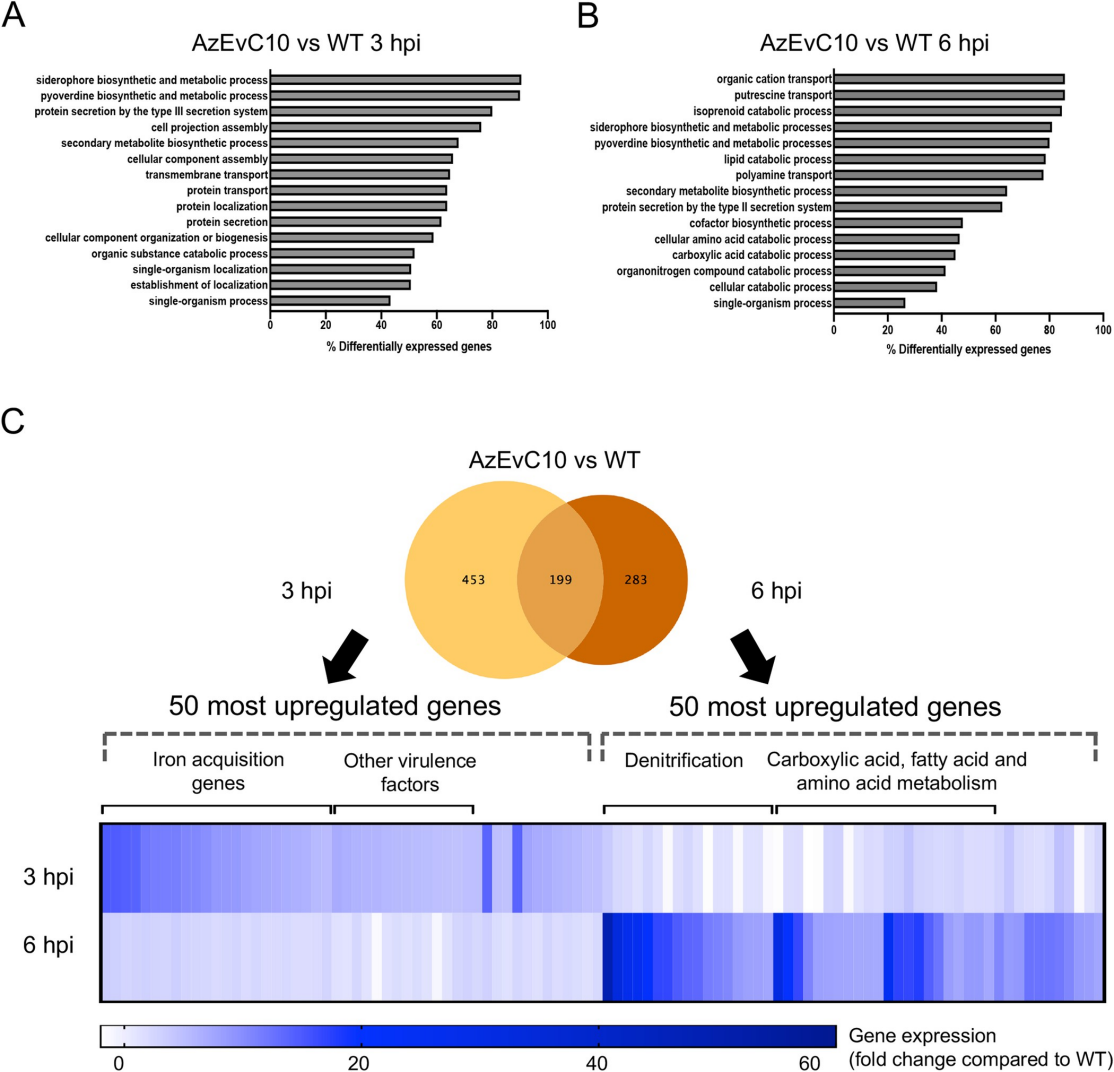
The AzEvC10 mutant upregulates virulence factor expression before metabolic gene expression during macrophage infection. **A-B.** Gene Ontology Enrichment Analyses reveal distinct enriched biological processes in the AzEvC10 mutant at 3 hpi (**A**) and 6 hpi (**B**). Percentage indicates relative number of genes in each pathway that were differentially regulated. **C.** Venn Diagram of AzEvC10 mutant DEGs compared to WT PAO1 at 3 hpi and 6 hpi of BMDM. The 50 most upregulated genes at each timepoint were selected and the expression is presented in mean fold-change compared to WT PAO1 (n = 2–5 independent replicates for each experiment).

It was unclear if the increased iron acquisition capacity in the AzEvC10 mutant preceded or followed its increased proliferation during BMDM infection. This distinction is important to resolve because either: 1) the increased capacity to capture iron could improve bacterial fitness under stressful conditions such as being in contact with host immune cells, or 2) increased mutant proliferation could create a greater need for iron and trigger the biosynthesis of pyochelin and pyoverdine siderophores. To resolve these two possibilities, we first performed Gene Ontology Enrichment Analyses to identify processes that were differentially regulated by the AzEvC10 mutant at 3 hpi and 6 hpi relative to WT infections. Our analyses revealed two distinct gene expression patterns between the two timepoints. At 3 hpi, the AzEvC10 mutant showed increased gene expression of siderophore biosynthesis and secretion processes, including siderophore and pyoverdine metabolic processes, T3SS, cell projection and assembly, protein membrane transport and secretion (Fig 4A). In contrast, at 6 hpi, genes in metabolic pathways such as isoprenoid, lipid, terpene carboxylic acid, and amino acid catabolism were differentially expressed (Fig 4B).

To confirm these findings, we compared AzEvC10 differentially expressed gene (DEG) sets at 3 hpi and 6 hpi and selected the 50 most upregulated genes at each timepoint (Fig 4C and S1 Table). At 3 hpi, 23 (46%) of the most upregulated genes were involved in pyoverdine biogenesis and ferric-siderophore import. Virulence factor secretion genes were also present in this gene set, with 13 (26%) upregulated genes including 10 genes involved in T3SS. At 6 hpi, 17 (34%) of the most upregulated genes were involved in denitrification processes. The other 24 (48%) genes were involved in carbohydrate, fatty acid and amino acid degradation, metabolism, and transport. These different signatures at 3 hpi and 6 hpi demonstrated how the phenotype of the AzEvC10 mutant changed during the BMDM infection. In the first hours of infection, AzEvC10 secreted pyochelin and pyoverdine siderophores as well as T3SS virulence factors and regulators. At 6 hpi, the gene expression signature shifted to a highly proliferative state and adaptation to decreased oxygen availability. Interestingly, operons involved in pyochelin biosynthesis *pchABCD* and *pchEFG*, as well as the transporter *fptA* were equally overexpressed at both 3 hpi and 6 hpi (S1 Table). These results support the hypothesis that the AzEvC10 mutant increased proliferation during BMDM infection due to a higher capacity to capture iron and by inducing more damage to the host cells by virulence factor secretion.

### Increased siderophore secretion results in ferroptosis in macrophages

**Fig 5:**
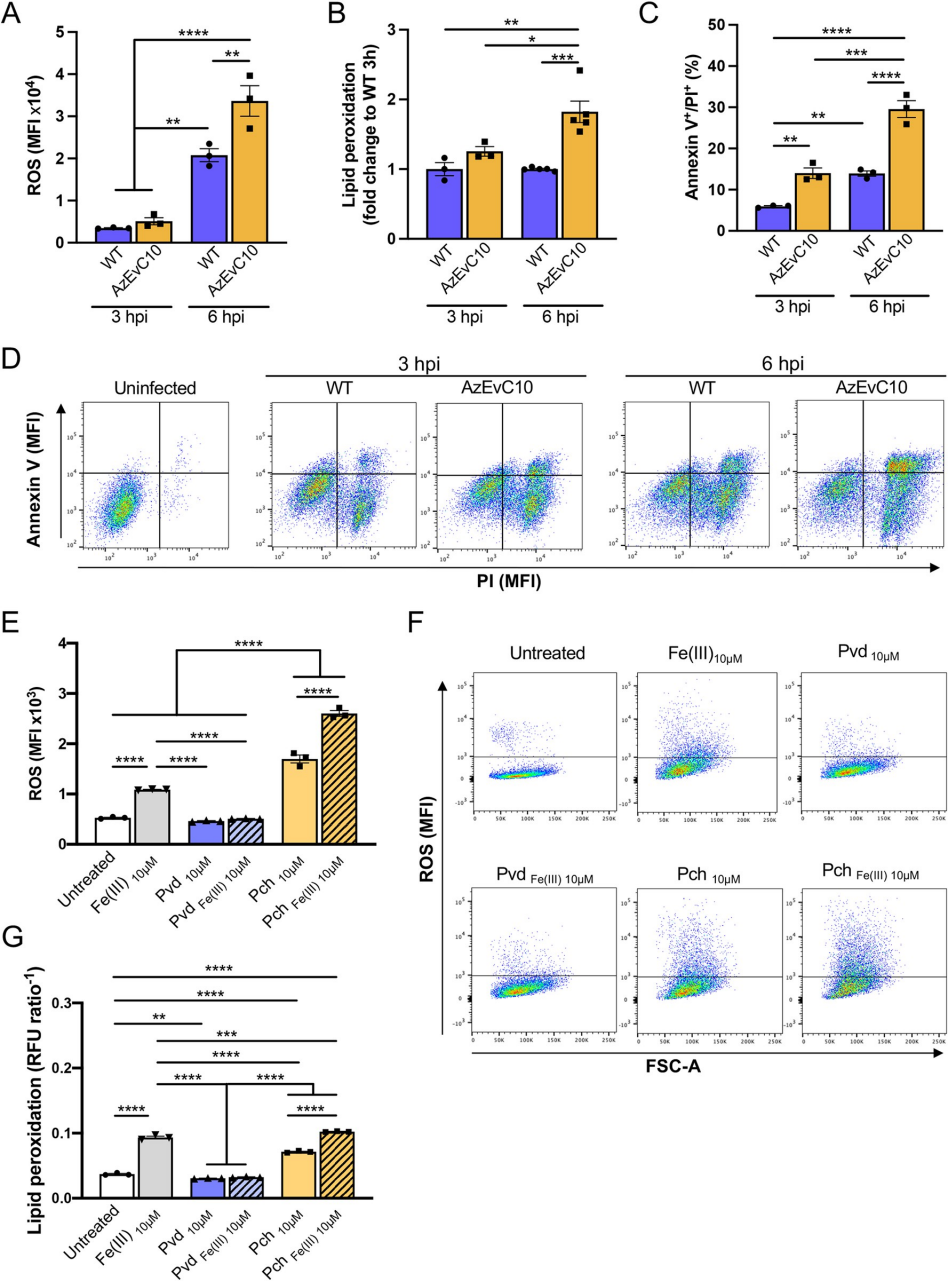
The AzEvC10 mutant induces iron dysregulation, oxidative stress, and ferroptosis in macrophages. BMDM cells were infected with WT PAO1 or AzEvC10 mutant at MOI:100 for 3 h and 6 h. **A.** Quantification of ROS in infected BMDM by flow cytometry. Quantification is presented in mean fluorescence intensity (MFI). Analysis was matched for experimental repeat. **B.** Quantification of lipid peroxidation in BMDM by flow cytometry presented as the reciprocal of the ratio of red (Ex561/Em582)/green (Ex488/Em525) fluorescence intensities normalized to WT PAO1 3 h. **C-D.** BMDM cell death was measured by flow cytometry and characterized by BMDM cells double positive for annexin V and PI. **E-G**. Uninfected BMDM were treated with 10 μM of either ferric iron (Fe(III)), pyoverdine (Pvd), ferric-pyoverdine (Pvd_Fe(III)_), pyochelin (Pch), or ferric-pyochelin (Pch_Fe(III)_) for 6h. Quantification of ROS presented in mean fluorescence intensity (MFI). **(E-F).** Quantification of lipid peroxidation in BMDM by flow cytometry presented as the reciprocal of the ratio of red (Ex561/Em582)/green (Ex488/Em525) fluorescence intensities **(G).** n = 3–6 independent replicates for each experiment. *p<0.05, **p<0.01, ***p<0.001, ****p<0.0001. See S5 Table for statistical tests used and exact *p*-values.

Reactive oxygen species (ROS) production is a defensive mechanism used by host cells to kill intracellular pathogens. However, too much ROS can cause host damage and lead to cell death. Therefore, we quantified general ROS production in BMDM during bacterial infection (Fig 5A). As expected, both WT and AzEvC10 infected BMDM increased their production of ROS at 6 hpi (*p*<0.01 and *p*<0.0001 compared to 3 hpi, respectively). However, BMDM infected with the AzEvC10 mutant had significantly greater ROS production at 6 hpi compared to macrophages infected with WT PAO1 (*p*<0.01).

We then assessed if this degree of ROS stimulated by infection with the AzEvC10 mutant was sufficient to induce cell damage. One of the consequences of ROS toxicity in cells is lipid peroxidation, in which free radicals attack double bonds of fatty acids leading to oxidative damage of polyunsaturated fatty acids and cell structure damage [31]. We used a ratiometric sensor to measure lipid peroxidation in BMDM and found that lipid peroxidation was significantly greater in AzEvC10 infected BMDM compared to those infected with WT PAO1 (*p*<0.05) (Fig 5B). Consistent with this, we found that BMDM infected with AzEvC10 exhibited high levels of annexin V and propidium iodide staining (Figs 5C–5D and S4A). The increase of these programmed cell death markers matched the increase in AzEvC10-mediated macrophage death shown in Fig 1B.

High ROS production and lipid peroxidation are hallmarks of ferroptosis, a form of iron-mediated programmed cell death. During bacterial infection, macrophages use different mechanisms to sequester iron and restrict its use by bacteria thereby limiting their pathogenicity [32]. However, iron overload can have deleterious effects on macrophages and impair their bactericidal functions. To determine if there was iron dysregulation in macrophages infected by the AzEvC10 mutant, we quantified the labile iron content in BMDM using a colorimetric method described by Abbasi et al. [33]. Since the labile iron content was similar in BMDM infected with the two strains(S4B Fig), a dysregulation in labile iron content was ruled out as a cause of the increased lipid peroxidation observed in AzEvC10-infected BMDM.

We then hypothesized that bacterial siderophores may be directly involved in the ROS production and/or lipid peroxidation in BMDM. In that sense, it has been shown that ferric-pyochelin could be an efficient catalyst for hydroxyl radical formation and induced cell injury [34,35]. Pyoverdine was also recently shown to translocate into host cells and disrupt iron mitochondrial homeostasis [36]. To test this hypothesis, we added 10 μM or 100 μM ferric iron (Fe(III)), pyochelin, ferric-pyochelin, pyoverdine or ferric-pyoverdine to healthy uninfected BMDM for 6 h. We then measured ROS production and lipid peroxidation in these cells. Interestingly, 10 μM pyochelin was sufficient to induce ROS production and lipid peroxidation at the same level as the Fe(III), and ROS production was even greater when BMDM were treated with 10 μM ferric-pyochelin (Fig 5E–5G). At 100 μM, Fe(III) and pyochelin did not induce more ROS and lipid peroxidation than the lower concentration, while 100 μM of ferric-pyochelin resulted in ROS production and lipid peroxidation exceeding levels of detection (S4C–S4E Fig). On the contrary, neither pyoverdine nor ferric-pyoverdine induced ROS or lipid peroxidation at both concentrations (Figs 5E–5G and S4C–S4E).

Although it is possible that other programmed or non-programmed cell death pathways (pyroptosis, necroptosis, necrosis, etc.) are also involved in BMDM death, together our results suggest that the increased pyochelin produced by the AzEvC10 mutant during infection induce excessive ROS and lipid peroxidation leading to ferroptosis of macrophages.

### Gallium treatment efficiently kills multidrug-resistant and hypervirulent *P*. *aeruginosa*

**Fig 6:**
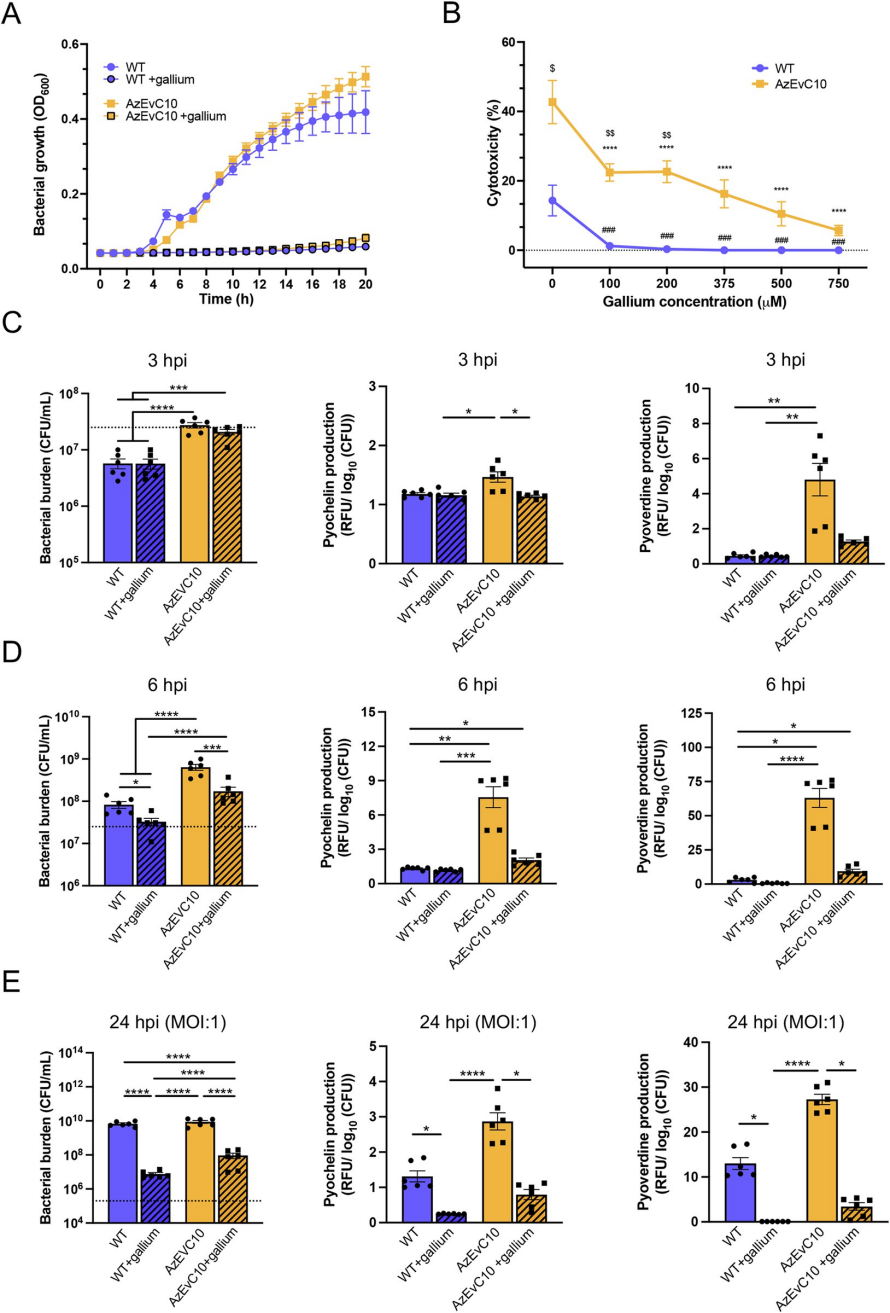
Gallium efficiently inhibits AzEvC10 siderophore production and proliferation during macrophage infection. **A.** WT PAO1 and AzEvC10 mutant growth curves in LB broth in the presence or absence of 150 μM gallium. **B.** BMDM were infected with either WT PAO1 or the AzEvC10 mutant (MOI:100) and treated with different concentrations of gallium for 6 h. BMDM cytotoxicity assessed by LDH assay: #represents the comparisons between WT with gallium to the WT no gallium baseline; *represents the comparisons between AzEvC10 with gallium to AzEvC10 no gallium baseline; $represents the comparisons between AzEvC10 and WT at the same gallium concentration. **C-D.** BMDM were infected with either WT PAO1 or the AzEvC10 mutant (MOI:100) and treated with 750 μM gallium for 3 h **(C)** and 6 h **(D)**. Bacterial burden was determined by viable CFU plate counts. Dotted lines represent the initial infection inoculum, 2.5 x 10^7^ CFU/mL. Pyochelin and pyoverdine production was measured by fluorescence (Ex350/Em430 for pyochelin and Ex400/Em460 for pyoverdine) normalized to log_10_(CFU). **E.** BMDM were infected with either WT PAO1 or AzEvC10 mutant (MOI:1) and treated with 750 μM gallium for 24 h. Bacterial burden was determined by viable CFU plate counts. Dotted lines represent the initial infection inoculum, 2.5 x 10^5^ CFU/mL. Pyochelin and pyoverdine production was measured by fluorescence (Ex350/Em430 for pyochelin and Ex400/Em460 for pyoverdine) normalized to log_10_(CFU). n = 6 independent replicates for each experiment. *p<0.05, **p<0.01, ***p<0.001, ****p<0.0001. See S5 Table for statistical tests used and exact *p*-values.

Gallium is an ion with similar properties to iron and has been proposed as an antimicrobial agent against *P*. *aeruginosa* [37]. Because virulence of the AzEvC10 mutant included a higher capacity to acquire iron and induce toxicity with siderophores, we sought to test if gallium was efficient against this multidrug-resistant mutant during BMDM infection. We first confirmed inhibition of bacterial growth with 150 μM gallium in LB broth (Fig 6A). We then tested gallium treatment during BMDM infection (Fig 6B–6E). As shown above (Fig 1B), the AzEvC10 mutant was markedly more toxic to macrophages than WT PAO1. Although gallium significantly decreased BMDM cytotoxicity caused by either strain in a dose-dependent manner (Fig 6B), we saw a significant difference in the protective effectiveness of this iron-like ion. Whereas cytotoxicity caused by WT PAO1 was completely abolished with the lowest dose of gallium (100 μM), BMDM death caused by AzEvC10 remained significantly elevated at all doses. We performed our next experiments using 750 μM gallium since this concentration was effective against the AzEvC10 mutant (Fig 6B) without significant toxicity in the BMDM (S5A Fig). Interestingly, at 750 μM gallium pyochelin and pyoverdine levels were significantly decreased in the AzEvC10 mutant without impacting bacterial growth at 3 hpi (Figs 6C and S5B). Siderophore production was still inhibited at 6 hpi but was accompanied by significant inhibition (~2.5–3.5-fold) of bacterial growth (Figs 6D and S5B).

We then infected BMDM for 24 h using a lower MOI (MOI:1) with or without gallium. Gallium was efficient at killing both WT (~1000-fold decrease, *p*<0.001) and the AzEvC10 mutant (~100-fold decrease, *p*<0.0001) *P*. *aeruginosa* (Fig 6E). As shown at the higher MOI, production of pyochelin and pyoverdine was greater in the AzEvC10 mutant compared to WT PAO1 (*p*<0.0001) and both were abolished with gallium at 24 hpi (Figs 6E and S5B). It is noteworthy that pyochelin and pyoverdine display a greater fluorescence when bound to gallium than the metal-free forms (S3A–S3D Fig) [30,38]. Thus, the fluorescence detected in the gallium treatment conditions may overestimate the real concentrations of the two siderophores.

Since gallium decreased siderophore production and bacterial load, we wanted to further clarify whether it had a direct impact on macrophage ferroptosis. In that sense, gallium could only act by blocking iron intake by the bacteria, leading to their death, or it could have additional direct protective effects on BMDM. To test this, we measured ROS production and lipid peroxidation in infected BMDM with or without gallium (S5C–S5H Fig). Interestingly, gallium slightly increased lipid peroxidation in BMDM at 3 hpi without any increase in ROS production (S5C and S5D Fig), which suggests modest toxicity in BMDM. Although this increase was significant, it was relatively stable over time (S5E–S5H Fig). Moreover, infected BMDM treated with gallium had lower ROS production and lipid peroxidation compared to untreated infected BMDM at 6 hpi and 24 hpi (S5E–S5H). This supports a protective role for gallium during bacterial infection that could outweigh its toxicity. Finally, we found that gallium-saturated pyochelin caused significantly less damage to BMDM compared to equivalent ferric-pyochelin concentrations (S5I and S5J Fig).

Altogether, our results suggest that gallium 1) has bactericidal potential against multidrug-resistant and hypervirulent *P*. *aeruginosa* strains, including strains like the AzEvC10 mutant, and 2) protects BMDM against ferroptosis during infection by counteracting ferric-pyochelin toxicity.

### Virulence and metabolic adaptation are common features of pulmonary infections

*P*. *aeruginosa* is a versatile bacterium that adapts to different environments. While the *in vitro* BMDM infections recapitulate interactions between *P*. *aeruginosa* and macrophages during *in vivo* infections, this model lacks several key environmental characteristics that mirror real-world human infections. These missing features include other host cells like neutrophils and epithelial cells, as well as the physical and chemical properties of sputum in CF airways. Thus, gene expression measured during *in vitro* experiments may not always accurately predict bacterial functioning during clinical infections.

**Fig 7:**
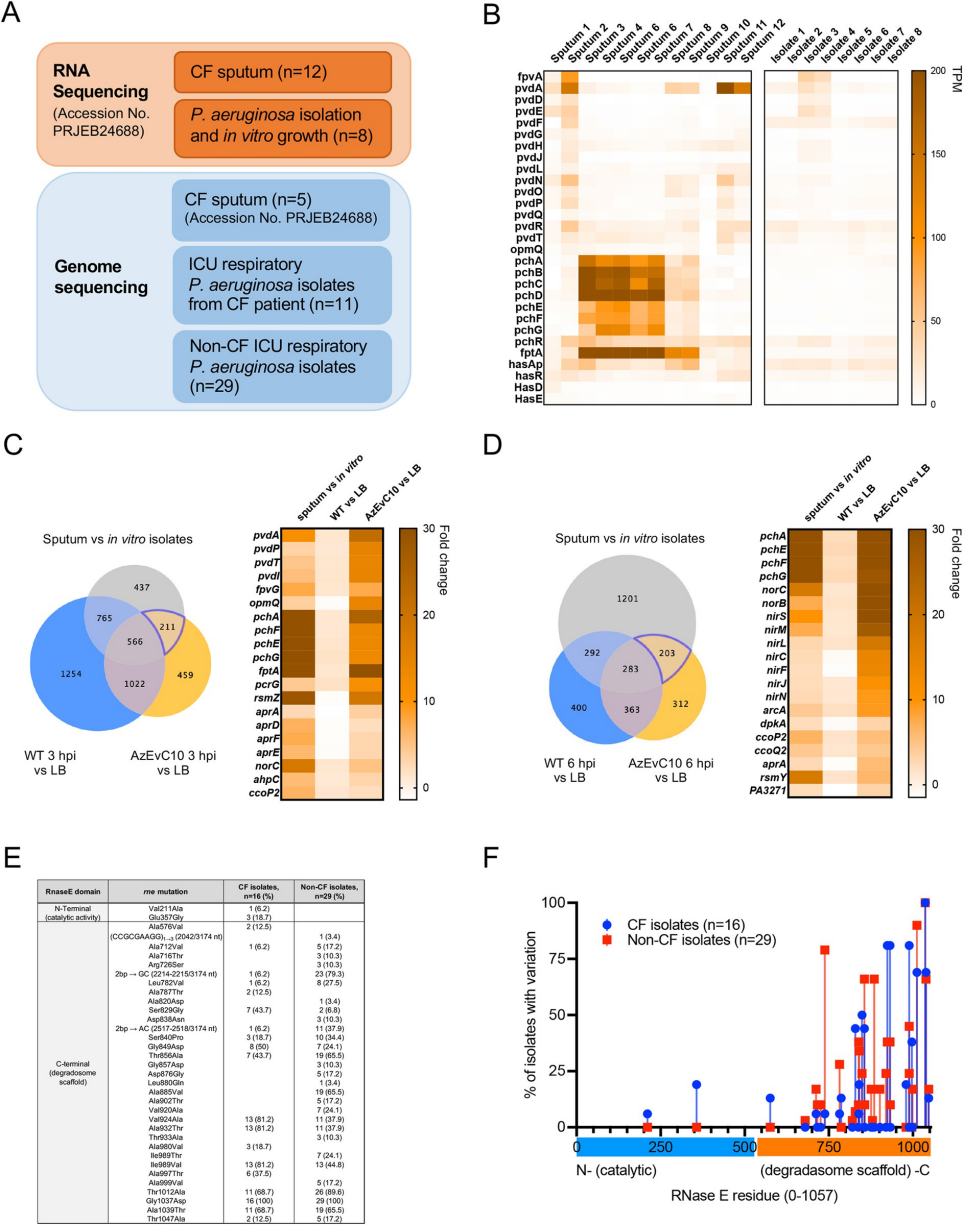
Virulence and metabolic adaptation are common features of *P*. *aeruginosa* pulmonary infections. **A.** Schematic representation of the experimental design for RNA and whole genome sequencing of *P*. *aeruginosa* clinical isolates. **B.** Individual expression of iron acquisition genes in CF sputum (n = 12) and *in vitro* grown clinical isolates (n = 8) expressed in normalized number of reads (TPM) **C-D.** Expression of selected iron and metabolic genes commonly upregulated by clinical isolates in sputum and by the AzEvC10 mutant during BMDM infection at 3 hpi **(C)** and 6 hpi **(D)** (n = 2–5). **E.** List of non-synonymous mutations detected in the *rne* gene in *P*. *aeruginosa* clinical isolates. **F.** Representation of the genomic location of *rne* mutations in clinical isolates. Fisher’s exact test was used to compare the prevalence of mutations in amino-terminal vs carboxy-terminal halves (*p*<0.0001) (S5 Table).

To test if the pathoadaptive phenotype observed in the AzEvC10 mutant during macrophage infection was reflective of *P*. *aeruginosa* gene expression profiles during real-world infections, we analyzed RNA-sequencing data from CF sputum and subsequently isolated *P*. *aeruginosa* clinical isolates grown *in vitro* [39] (Fig 7A). In accordance with published findings [39], our analyses showed that pyochelin biosynthesis enzymes (*pchABCD*, *pchEFG*) and its transporter (*fptA*) were significantly overexpressed during pulmonary infections of CF patients but not when grown *in vitro* (Fig 7B and S2 Table). We then compared the genes that were differentially expressed between the sputum samples and *in vitro* grown *P*. *aeruginosa* with the DEGs of our WT PAO1 or AzEvC10 strains between macrophage infection and LB growth. As expected, common upregulated genes between AzEvC10 mutant 3 hpi and sputum included pyoverdine and pyochelin biosynthesis enzymes and transporters (Fig 7C and S2 Table). Other common genes were involved in the response to oxidative stress (*ahpC*), T1SS (*aprA*, *aprFED*), and regulation of the T3SS (*rsmZ* and *pcrG*, respectively). Interestingly, expression of the alkaline protease (*aprA*) and its secretory apparatus (*aprFED*) is regulated by iron levels and they facilitate iron acquisition by the proteolytic cleavage of transferrin [40,41]. This highlights the importance of iron acquisition during infection and a role for *rne* mutants in iron-mediated bacterial virulence. A subset of commonly upregulated genes shared by the AzEvC10 mutant 6 hpi and sputum *P*. *aeruginosa* are known to be expressed under anaerobic conditions, including the *nir* and *nor* operons, cytochrome C oxidase subunits (*ccoP2* and *ccoQ2*), arginine deaminase (*arcA*), and malate/L-lactate dehydrogenase (*dpkA*) (Fig 7D and S2 Table). This switch to anaerobic metabolism contributes to increased fitness of *P*. *aeruginosa* under hypoxic conditions. The open reading frame PA3271 was also overexpressed in both AzEvC10 at 6 hpi and sputum and encodes a probable two-component sensor. Because more than 50% of two-component systems have been implicated in virulence and *in vivo* fitness and colonization ability [15], this suggests that the sensor may be important for *P*. *aeruginosa* responses to macrophages. Hence, our results show that the pathoadaptive changes seen in AzEvC10 during macrophage infection share similarities with *P*. *aeruginosa* behavior during pulmonary infections.

### Clinical isolates evolve multiple mutations in the *rne* gene

The *rne* gene encodes RNase E endonuclease, a 1,057-residue protein involved in RNA processing and decay [42]. Because the virulent phenotype of AzEvC10 mutant is caused by a 50-bp deletion in the of 3’ end of the *rne* gene encoding the enzyme’s carboxy-terminus, we assessed if mutations in this region were present in human clinical isolates. We performed whole genome sequencing on *P*. *aeruginosa* isolates from both CF (n = 16) and non-CF (n = 29) respiratory infections (Fig 7A). We found that all isolates (n = 45) had multiple non-synonymous mutations in the *rne* gene (Fig 7E). All non-synonymous mutations except for two were found in the second half of the gene, which encodes the carboxy-terminal half of the RNase E protein (*p*<0.0001) (Fig 7F). Based on our observations in the AzEvC10 mutant during macrophage infection, these results suggest that *rne* variations evolved in *P*. *aeruginosa* clinical isolates may have contributed to their colonization and/or fitness during pulmonary infections. However, because clinical isolates likely have additional mutations in other genes, it is difficult to directly demonstrate the role of *rne* mutations on the gene expression and fitness of these clinical isolates. Further investigation will be needed to determine which variants affect RNase E functions and bacterial phenotypes during infection.

## Discussion

*P*. *aeruginosa* is present in more than 50% of adults with CF and is thought to adapt to the CF lung environment by decreasing production of virulence factors and acquiring antibiotic resistance features. In this study, we challenge this concept of an inverse correlation between acquisition of antibiotic resistance and virulence factor production. In a previous study, we showed that aztreonam selected for mutations of the MexAB-OprM regulator genes, including *nalD* [26]. Mutants carrying mutations in the *nalD* gene were multidrug resistant and found in 7–33% of clinical isolates, depending on the study [26,43–47]. In this study, we showed that the multidrug-resistant AzEvC10 mutant evolved a compensatory mutation in the *rne* gene, which led to increased survival and increased proliferation during macrophage infection compared to WT and induced significantly higher cytotoxicity in macrophages (Figs 1 and S1). Increased macrophage killing *in vivo* could lead to impaired host protection against pathogens and exacerbated inflammation [4,5,48–52]. We showed that although aztreonam resistance was due to a loss-of-function *nalD* mutation, the AzEvC10 hypervirulent phenotype resulted from a *rne* gene variant (Figs 2 and S2). The evolution of increased virulence towards macrophages was surprising, given that this phenotype evolved only in response to aztreonam, suggesting that antibiotic selection alone can lead to hypervirulent phenotypes.

RNase E is an endonuclease with two main domains: a catalytic amino-terminus, and a carboxy-terminus which serves as a scaffold for the degradosome complex [53,54]. In *E*. *coli* the last residues of C-terminus contain the binding site for the exoribonuclease Polynucleotide phosphorylase (PNPase) [54]. Thus, a 17-residue deletion in that region as seen in the AzEvC10 mutant could prevent or impair PNPase binding and overall degradosome activity. Although the absence of the degradosome complex enhanced RNA half-lives in *E*. *coli*, some studies suggested that PNPase was unnecessary for RNA degradation and was rather a scavenger of RNA intermediates [42,55–57]. However, mutants lacking PNPase or the PNPase-binding site in RNase E were sufficient to increase RNA half-lives of specific mRNAs, suggesting that the presence of PNPase in the degradosome complex is necessary for degradation of specific mRNAs [58]. In our study, a 17-residue deletion in the PNPase-binding site of RNase E was sufficient to create a global transcriptomic shift in the AzEvC10 mutant during macrophage infection. We found a striking upregulation of pyoverdine and pyochelin secretion by AzEvC10, *rne*_Δ50bp_, and *rne*::Tn strains (Figs 3 and S3E and S1 Table). Interestingly, heme sensor genes of the Has system were also upregulated, but not those involved in the Phu system (Fig 3 and S1 Table). This suggests that the mutation in AzEvC10 RNase E impacts specific substrates as opposed to the whole mRNA pool. Furthermore, the AzEvC10 mutant overexpressed pyochelin and pyoverdine in the presence of macrophages (Figs 3 and S3E), but not in LB medium (Fig 3B) or in the macrophage growth medium only (S3F Fig). These results suggest that both siderophores are still regulated at the transcriptional level, and that *rne* mutation may lead to a stabilization of the pyochelin and pyoverdine pathway mRNAs. *E*. *coli* RNase E was also shown to interact with RNA-binding proteins such as Hfq in a degradosome-independent manner [56,59,60]. However, the literature differs on the necessity of this interaction for RNA cleavage [56,59,61]. The RNase E carboxy-terminal region was also shown to be essential for binding and localization of the degradosome to the bacterial membrane, which could affect its activity [62,63]. In *P*. *aeruginosa* in general, and in our study, it is unknown whether the RNase E-Hfq interaction or cellular localization are impaired in the AzEvC10 mutant. However, due to the location of the AzEvC10 RNase E mutation, we believe this to be unlikely. Ultimately, future work will be necessary to determine the precise effects of the RNase E C-terminal variation on its biochemical functions.

The transcriptional upregulation of iron acquisition pathways preceded the metabolic changes (Fig 4 and S1 Table), suggesting that this increased virulence and capacity to capture iron drove the proliferative phenotype of the AzEvC10 mutant during macrophage infection. Siderophore production gene transcription is regulated by iron starvation however, their secretion can also have additional effects other than iron acquisition. For instance, pyochelin can increase *P*. *aeruginosa* competitiveness against other bacteria and cause tissue and host cell injury by catalyzing the formation of ROS via the Fenton reaction [34,35,64]. On the other side, bacteria protect themselves from ROS by upregulating enzymes involved in superoxide metabolism (*ahpC*, *sodM*) (S1 Table). The T3SS can also induce macrophage lysis [9,10] and was previously shown to be positively regulated by RNase E in *P*. *aeruginosa* [65]. Although T3SS genes were upregulated at 3 hpi by the AzEvC10 mutant, their negative regulators, the *rsmY* and *rsmZ* small non-coding RNAs were upregulated as well (Fig 4 and S1 Table). Therefore, it is not clear whether the T3SS is active or inactive in the AzEvC10 strain. Future studies will examine the role of this virulence system in the host cell damage and bacterial escape using different *rne* mutants. RNase E or homologs were also involved in bacterial virulence and metabolic adaptation under stress conditions in different bacterial species [56,60,66,67]. We speculate that the stress induced by phagocytosis triggers the secretion of virulence factors by the bacteria to survive the phagocytic process and eliminate a threat.

The AzEvC10 mutant significantly increased cell toxicity by inducing excessive ROS in macrophages, lipid peroxidation, and ferroptosis (Figs 5 and S4). While other cell death pathways, like pyroptosis or necroptosis, may also be involved in the BMDM cell death, the lipid peroxidation is a hallmark of ferroptosis [68]. This was somewhat unexpected, as macrophages activated with LPS express iNOS to resist ferroptosis triggered by chemical treatment with the pro-ferroptotic GPX4 inhibitor, RSL3 (1S,3R)-2-(2-chloroacetyl)-2,3,4,9-tetra-hydro-1-[4-(methoxycarbonyl)phenyl]-1H-pyrido[3,4-b]indole-3-carboxylic acid) [69]. However, in our RNA-seq analyses we observed no decrease in iNOS expression (not shown) in the mutant infected BMDM, yet lipid peroxidation was still prevalent, indicating that activated macrophages may be susceptible to ferroptosis during infection. This apparent discrepancy between the mutant infected BMDM and LPS activated BMDM could be explained by the competition between the host and microbe for iron. Macrophages respond to bacterial infection by sequestering iron, which prevents its use by pathogens. In physiological conditions (pH 7.4), transferrin has a greater affinity for iron (10^20^ M^-1^) than pyochelin (10^18^ M^-1^), while pyoverdine has the highest affinity for iron (10^32^ M^-1^) [30,70]. Both transferrin and pyochelin were shown to have the capacity to steal iron from each other in physiological conditions, although pyochelin and pyoverdine capacity to acquire iron from transferrin is optimal at lower pH [71–73]. It was previously shown that ferric-pyochelin was an efficient catalyst for hydroxyl radical formation and induced cell injury [34,35]. Pyoverdine was also recently shown to translocate into host cells and disrupt iron mitochondrial homeostasis [36]. In our study, ferric-pyochelin, but not ferric-pyoverdine, was sufficient to induce exacerbated ROS production and lipid peroxidation in healthy macrophages (Figs 5E–5G and S4C–S4E). Metal-free pyochelin was also able to induce ROS and lipid peroxidation to some extent (Figs 5E–5G and S4C-S4E). Interestingly, increasing the pyochelin concentration from 10 μM to 100 μM did not result in increased ROS production and lipid peroxidation. This may suggest that 1) the effect of metal-free pyochelin is minimal and easily restrained by macrophages, or 2) metal-free pyochelin itself has no effect, but a certain proportion of this pyochelin binds iron in the macrophage growth medium, and it is this ferric-pyochelin that drives the ROS and lipid peroxidation detected in macrophages. In that sense, increasing the concentration of pyochelin does not result in increased effect since in this condition, the effect is limited by the iron content in the growth medium.

Lipoxygenases and cyclooxygenases can also oxidize lipids. It was recently demonstrated that some biofilm-producing *P*. *aeruginosa* isolates overexpressed the enzyme pLoxA, which induced ferroptosis in human bronchial epithelial cells by oxidation of arachidonic acid–phosphatidylethanolamines [74]. In our study, pLoxA was not increased in the AzEvC10 mutant. Although this enzyme is involved in cell lipid oxidation, it is unlikely the root cause of increased lipid peroxidation and subsequent ferroptosis observed in the macrophages here. Lipid peroxidation is a hallmark of CF [75,76] and ferroptosis was recently described in CF epithelial cells [77–79]. Our study expands the current understanding of how *P*. *aeruginosa* can induce ferroptosis in CF, showing that 1) multidrug-resistant *P*. *aeruginosa* can induce ferroptosis in macrophages, one of the most important cell types in bacterial clearance [4–7], and 2) RNase E variants can confer bacterial hypervirulence by triggering host cell ferroptosis. Since both ferroptosis and dysfunctional macrophages lead to exacerbated inflammation and tissue damage, it is imperative to develop therapeutics that will target this process and protect host cells. One of such treatment could be the use of ferroptosis inhibitors to prevent cell death. Another approach, the one chosen in the current study, is to target the root cause of ferroptosis, bacterial siderophores, by using iron competitors.

Since siderophores were increased in the AzEvC10 mutant and induced toxicity during macrophage infection, we sought to target and inhibit these molecules using gallium nitrate. Previous work suggested gallium as a novel therapy for its antimicrobial properties against *P*. *aeruginosa* and *Klebsiella pneumoniae* [37,80–85]. In the present study, gallium significantly decreased siderophore-mediated cytotoxicity in BMDM (Figs 6 and S5). Moreover, gallium treatment for 24 h decreased bacterial growth by a factor of 100- to 1000-fold. This is important since AzEvC10 mutant *P*. *aeruginosa* is resistant to multiple antibiotics currently used in CF patients [26]. A first clinical trial conducted by Goss *et al*. showed gallium to be a suitable treatment for CF patients [37]. However, a larger study (NCT02354859) showed less promising results, although gallium did slightly improve respiratory symptoms and decreased bacterial density in sputum cultures. Different hypotheses may explain this lack of efficiency of gallium treatment seen in CF patients. One of them is the low concentration of gallium detected in the sputum, likely caused by the intravenous administration of the treatment [37]. To tackle this issue, inhalation of gallium was suggested as a potentially more effective mode of administration compared to intravenous delivery in a pre-clinical model [86]. As a result, a clinical study is currently ongoing to test the safety and pharmacokinetic properties of inhaled gallium in CF patients (NCT03669614). A second hypothesis is that the people treated with gallium were infected with *P*. *aeruginosa* that would not be affected by the gallium treatment. Our data suggest that gallium could be an effective precision treatment for people infected with *P*. *aeruginosa* strains that overexpress siderophores. Finally, the experimental design used in our study is a model of acute infection. This leads us to believe that gallium treatments could be more effective in acute rather than chronic respiratory infections by *P*. *aeruginosa*.

An important aspect when considering treatments with gallium (or any compound) is the potential intrinsic toxicity of such compounds to the host. In human plasma, malondialdehyde, a product of lipid peroxidation, was positively correlated with the levels of urinary gallium in exposed electronic industry workers [87]. In our study, we found that gallium had a low toxicity in BMDM at up to 1 mM (S5A Fig). Although 750 μM treatments resulted in a slight but significant increase in lipid peroxidation in BMDM, this side effect was stable over time and lower than what was measured in untreated infected macrophages (S5C–S5H Fig). Furthermore, gallium treatment inhibited ROS overproduction in infected BMDM (S5C, S5E and S5G Fig), supporting gallium as a beneficial treatment during infections. We also showed that gallium-saturated pyochelin was also much less toxic than ferric-pyochelin, decreasing macrophage ferroptosis (S5I–S5K Fig). However, gallium-bound pyochelin was also more toxic than the metal-free pyochelin (S5I–S5K Fig), which could raise some concerns for *in vivo* treatments. But since gallium was well tolerated in pre-clinical and clinical trials [37], we believe that gallium antimicrobial properties may overcome potential toxic side effects during bacterial infections. Finally, local (inhaled) instead of systemic (intravenous) gallium treatments may restrain other potential side effects.

Increased iron acquisition, resistance to oxidative stress and changes in metabolism were detected in *P*. *aeruginosa* in sputum and in the AzEvC10 mutant during macrophage infection (Fig 7). These differentially expressed pathways were previously described in a very elegant study by Rossi *et al*. [39]. The comparison between our experimental model and clinical isolates from CF sputum support our findings by showing that phenotypes detected in the AzEvC10 mutant are also detected during infection. Furthermore, we found a striking abundance of variations in the carboxy-terminus of the *rne* gene in *P*. *aeruginosa* clinical isolates (Fig 7), suggesting that *rne* variations evolved in *P*. *aeruginosa* clinical isolates could enhance their colonization and/or fitness during pulmonary infections. Comparing an evolved lab strain with clinical isolates does have limitations. An obvious limitation is that the accumulation of genetic mutations in these clinical isolates during CF chronic infection could have secondary effects on the phenotypes that might be induced by RNase E variations. Clinical isolates from CF sputum were shown to have distinct genetic backgrounds [39]. These differences could impact genes involved in iron acquisition, resistance to oxidative stress, or metabolic pathways. Therefore, the direct effects of *rne* mutations on these pathways are difficult to directly demonstrate without in-depth future investigation.

Some limitations exist in our study. We speculated that the degradosome assembly and/or function was impaired in the AzEvC10 mutant, explaining its increased virulence by siderophore overexpression. However, we have not directly tested the effects of the *rne* mutation on degradosome function, nor the interaction of RNase E with PNPase. The mutation could also impair RNase E interaction with other proteins, like RNA-binding proteins, rather than PNPase. These aspects will need to be elucidated in further studies. Finally, we used healthy instead of CF macrophages for infections. It was previously shown that a dysfunctional cystic fibrosis transmembrane conductance regulator (CFTR) channel sensitized epithelial cells to lipid peroxidation and ferroptosis [77]. However, CFTR corrector only partially reversed lipid peroxidation [79]. Furthermore, *P*. *aeruginosa* equally caused lipid peroxidation and ferroptosis in the airways of CFTR^+/+^ and CFTR^-/-^ mice and in human airway epithelial cells expressing WT CFTR or F508Del mutant CFTR [78]. Thus, it is unclear whether *P*. *aeruginosa* overexpressing siderophores would display a greater toxicity to macrophages expressing a dysfunctional CFTR channel.

Our study underscores how a second-site compensatory mutation evolved during antibiotic selection can restore virulence in multidrug-resistant *P*. *aeruginosa*. Furthermore, we demonstrate a role for RNase E endonuclease in bacterial virulence and metabolic adaptation during infection. We also showed that mutations in the *rne* gene are prevalent in clinical isolates from acute and chronic pulmonary infections. Second-site mutations, including mutations in the *rne* gene, could be a key mechanism that lead multidrug- and extensively drug-resistant *P*. *aeruginosa* to be hypervirulent in CF. Because infections with resistant *P*. *aeruginosa* are increasing worldwide [20–22], the need for new therapies is more urgent than ever. Gallium nitrate is a promising therapy for these “superbugs” and targeting ferroptosis may represent another means to reduce *P*. *aeruginosa* pathogenesis.

## Materials and methods

### Bacterial strains and growth conditions

Bacterial strains and plasmids are listed in S3 Table. *P*. *aeruginosa* PAO1 AzEvC10 and AzEvC10 pMQ72::*nalD* mutants were obtained from Pradeep K. Singh [26]. The *rne* Tn mutant (*rne*::Tn) was obtained from Colin Manoil’s laboratory at the University of Washington and corresponds to the PW5993 mutant containing the genotype PA2976H05:: IS*phoA*/hah [29]. This mutant was grown from freezer stock on Lysogeny broth (LB) agar (BD, cat# 244520) with 10 mg/mL tetracycline. All strains were maintained at 37°C in LB (BD, cat# 244620) unless otherwise specified. Where necessary, gentamicin (Gm) was added to growth medium at the following concentrations: 10 μg/mL for *E*. *coli* and 30 μg/mL for *P*. *aeruginosa*.

### *P*. *aeruginosa* clinical isolates

*P*. *aeruginosa* clinical isolates from CF (n = 11) or non-CF (n = 29) ICU patients with pulmonary infection were a generous gift from the Pulmonary Translational Research Core at University of Pittsburgh.

### Mutant constructions

Gene deletion mutants listed in S3 Table were generated with suicide plasmids as described previously [88]. Suicide plasmids carrying *mexAB* deletion, *nalD*_T158P_ point mutation, 50bp deletion in *rne* gene, or WT *rne* gene were constructed in a similar way. First, two PCR fragments were generated from chromosomal DNA using corresponding primer pairs to the up- and down-stream regions of each gene (see S4 Table for primer nucleotide sequences). The two fragments were then assembled into the suicide vector pEX18Gm between using NEBuilder HiFi DNA Assembly Cloning Kit (cat# E5520). Suicide plasmids were then transformed into *E*. *coli* DH5α according to the NEB protocol and verified by Sanger sequencing. *P*. *aeruginosa* strains were transformed by electroporation as published previously [89] and incubated on LB + Gm 30 μg/mL at 37°C overnight. For counter selection, isolated clones were streaked on low-salt LB containing 15% sucrose as published [88] and incubated at room temperature for 48 h. Gene deletions were confirmed by PCR using primers listed in S4 Table and whole genome sequencing.

### DNA extraction, purification, and PCR

Plasmid DNA was prepared using a Monarch Plasmid Miniprep Kit (NEB, cat# T1010). Genomic DNA was prepared using the DNeasy Blood & Tissue Kit (Qiagen, cat# 69504). When necessary, cDNA was purified using the Monarch PCR & DNA Cleanup Kit (NEB, cat# T1030). PCR was performed using either KAPA HIFI 2X ready mix (KAPA Biosystem, cat# KK2602) or Expand Long Template PCR System (Sigma, cat# 11681842001). Primers used for each PCR reaction are listed in S4 Table.

### Aztreonam susceptibility testing

Etest strips were purchased from Biomerieux Vitex Inc (cat# 501758) and antimicrobial susceptibility testing was performed with the following modifications to the manufacturer’s instructions. A sterile swab was soaked in an overnight culture for each strain after growth for 18h in LB broth and excess fluid was removed by pressing it against the inside wall of the test tube. Mueller Hinton agar plates were fully streaked 4 times with the swabs. After allowing the plates to dry, an Etest gradient strip was placed in the middle of each plate. Plates were incubated at 37°C for 16–20 h and MICs were read by zone of clearing around the strip.

### BMDM isolation and phenotype verification

Femurs and tibias from C57BL/6 mice were harvested and cleaned up with 70% ethanol. Working in a biological safety cabinet, bones were washed with 1X PBS. Bone marrow was flushed with 10 mL of BMDM growth medium pH 7.6 (RPMI 1640 containing 20% L929 conditioned medium, 10% fetal bovine serum [FBS, Omega Scientific cat# FB-11] and antibiotic-antimycotic). Antibiotic-antimycotic solution was purchased from ThermoFisher (cat# 15-240-062) and used at a final concentration of 1% (100 units/mL penicillin, 100μg/mL streptomycin, and 2.5 μg/mL fungizone). Red blood cells were lysed in 5 mL 1X RBC lysis buffer for 3 m at room temperature. Lysis was stopped by adding 30 mL of 1X PBS. Cells were pelleted, counted and plated on treated tissue culture dishes. After 24 h, non-adherent cells were plated into a petri dish and grown in BMDM growth medium at 37°C and 5% CO_2_. Fresh medium was added after 3 d. After 7 d, cells were plated for experiments. BMDM phenotypes were confirmed for their positivity for the alveolar macrophage markers CD64 and Siglec F. Briefly, cells were washed with 1X PBS and then harvested with 1mL 5mM EDTA-1X PBS and centrifuged at 8000rpm for 1 m. Supernatants were removed, and cell pellets were washed with 1 mL FC buffer (3% FBS in 1X PBS). BMDM were resuspended in 50 μL blocking buffer (1:25 FC block [BD cat# 553142] in FC buffer) and incubated on ice. After 20 m, 50 μL of the 2% FBS in 1X PBS containing 0.25 μL of the following antibodies was added and incubated on ice for 30 m: CD45 (Biolegend cat# 103137), CD64 (Biolegend cat# 139307) and Siglec F (BD cat# 562681). Cells were then washed in cold 1X PBS and resuspended in 300 μL 1X PBS for flow cytometry analysis.

### Bone marrow-derived neutrophil isolation

Femurs and tibias from C57BL/6 mice were harvested and cleaned up with 70% ethanol. Working in a biological safety cabinet, bones were washed with 1X PBS. Bone marrow was flushed with 10 mL of growth medium (RPMI 1640 containing 10% FBS). The EasySep Mouse Neutrophil Enrichment Kit (StemCell, cat# 19762) was used to isolate neutrophils according to the manufacturer’s instructions. Neutrophils were used the same day of isolation.

### Mouse airway epithelial cells

Immortalized mouse airway epithelial cells (MLE-12) were a gift from Dr. Peter Chen (Cedars-Sinai Medical Center, Los Angeles). Cells were grown in Dulbecco’s Modified Eagle Medium containing 10% FBS (Omega Scientific cat# FB-11).

### L929 conditioned medium

L929 mouse fibroblasts were grown in Iscove’s Modified Dulbecco’s Medium (ThermoFisher, cat# 12-440-061) with 10% FBS. Cells were plated at a density of 4 x10^6^ cells/150 mm tissue culture treated dish and culture for 10 d at 37°C, 5% CO_2_. Medium was then collected, filtered through a 0.22 μm filter and frozen at -80°C in 50 mL aliquots.

### Alveolar macrophage isolation and infection

Wild-type C57BL/6 mice were anesthetized using 4% isoflurane. Cardiac perfusion was performed with sterile 1X PBS. Then, the trachea was exposed and punctured, and a 20G intratracheal tube was inserted into the trachea. A 1mL syringe filled with sterile 2mM EDTA + 2% FBS in 1X PBS was attached to the tube and gently injected into the lungs. The volume was slowly aspirated and transferred on 15 mL tube on ice. This was repeated 7 additional times for a total of 8 mL. The BAL was centrifuged and resuspended in macrophage growth medium (RPMI 1640 containing 20% L929 conditioned medium and 10% fetal bovine serum [FBS, Omega Scientific cat# FB-11]). Cells were incubated overnight at 37°C and 5% CO_2_. The next day, alveolar macrophages were visually confirmed to be adherent. Alveolar macrophages were then infected with WT or AzEvC10 mutant *P*. *aeruginosa* MOI: 100 for 6 h and the bacterial loads were determined by CFU counts as described below.

### Cell infection with *P*. *aeruginosa*

BMDM, neutrophils, or AEC were plated with growth medium without antibiotic-antimycotic for all experiments. Bacterial strains were streaked on LB agar and incubated at 37°C. The day before the infection, bacterial strains were grown in LB broth at 37°C overnight shaking at 250rpm. The day of infection, strains were diluted and grown to mid-exponential phase OD_600_ 0.4–0.6. Bacteria were then pelleted, washed with 1X PBS and resuspended in 1X PBS. BMDM were infected with a MOI: 100 for 3 h or 6 h, or with a MOI: 1 for 24 h. For intracellular survival experiments, BMDM were infected for 1 h, then amikacin was added to a final concentration of 200 μg/mL and left for an addition 5 h of incubation. For the escape experiments, antibiotics were added after 1 h incubation and left for 1 h. Cells were then washed with 1X PBS and fresh BMDM medium without antibiotic was added and cells were incubated for a total of 3 h or 6 h.

### Immunofluorescence

BMDM were infected with a MOI: 100 for 6 h. Cells were then washed with 1X PBS and fixed with 2% PFA for 10 m at room temperature. After 3 washes with 1X PBS, cells were permeabilized in 1X PBS + 5% Goat serum + 0.1% Triton X-100 for 5 m at room temperature. Anti-CD68 (BioRad, cat#MCA1957, 1: 250) and anti-*P*. *aeruginosa* (ThermoFisher, cat# MA183430, 1:500) primary antibodies were diluted in 1X PBS + 2% Goat serum. Cells were incubated with primary antibodies for 1h at room temperature, washed 3 times with 1X PBS, then incubated for 30–45 m with goat anti-mouse Alexa Fluor 555 (Invitrogen cat#A32727, 1:1000) or goat anti-rat Alexa Fluor 488 (Invitrogen cat#A11006, 1:1000) secondary antibodies. After 3 washes with 1X PBS, cells were mounted with Fluoromount G mounting medium with DAPI (ThermoFisher, cat# 00-4959-52). Images were taken using a Zeiss Apotome fluorescence microscope and analyzed with Bitplane Imaris software version 9.2.1.

### Cytotoxicity assay

Cytotoxicity assays were performed using the Pierce LDH Cytotoxicity Assay Kit (ThermoFisher, cat# 88954) according to manufacturer’s instructions.

### Reactive oxygen species assay

Infected cells were washed with 1X PBS and then harvested with 1mL 5mM EDTA-1X PBS and centrifuged at 8000rpm for 1 m. Supernatants were removed, and cell pellets were washed with 1 mL FC buffer (3% FBS in 1X PBS). BMDM were resuspended in 10 μM 2′,7′-dichlorodihydrofluorescein diacetate dilution (Sigma cat# D6883) and incubated for 30 m at 37°C protected from light. Cells were then washed in 1 mL FC buffer and resuspended in FC buffer and immediately analyzed by flow cytometry (Becton Dickinson LSR Fortessa). Mean fluorescence intensity (MFI) was quantified using FlowJo software (version 10.6.1, FlowJo LLC).

### Lipid peroxidation assay

Infected cells were washed with 1X PBS and then harvested with 1 mL of 5 mM EDTA-1X PBS and centrifuged at 8000rpm for 1 m. Supernatant was removed, and cell pellets were washed with 1 mL FC buffer (3% FBS in 1X PBS). The Lipid Peroxidation Assay Kit (Abcam cat# ab243377) was used and cells were stained according to the manufacturer’s instructions. Cells were then washed in 1 mL Hank’s Balanced Salt Solution (HBSS) and resuspended in HBSS buffer and immediately analyzed by flow cytometry (Becton Dickinson LSR Fortessa). Mean fluorescence intensity (MFI) was quantified using FlowJo software (version 10.6.1, FlowJo LLC). Lipid peroxidation was quantified by calculating the red (Ex561/Em582)/green (Ex488/Em525) fluorescence ratio using FlowJo software (version 10.6.1, FlowJo LLC). Data are presented as the reciprocal of the ratio (1/ratio) and normalized on WT PAO1 at 3 h where specified.

### Cell death assay

Infected cells were washed with 1X PBS and then harvested with 1 mL of 5 mM EDTA-1X PBS and centrifuged at 8000rpm for 1 m. Supernatant was removed, and cell pellets were washed with 1 mL FC buffer (3% FBS in 1X PBS). To quantify cell death, TACS Annexin V-FITC Apoptosis Detection Kit (R&D Systems cat# 483001K) was used and cells were stained according to the manufacturer’s instructions. Cells were then washed with the binding buffer and resuspended in binding buffer and immediately analyzed by flow cytometry (Becton Dickinson LSR Fortessa). The percentage of double positive cells and MFI were quantified using FlowJo software (version 10.6.1, FlowJo LLC).

### CFU counts to determine total and intracellular bacterial burdens

BMDM were permeabilized with 0.1% Triton X-100 for 5 m, then cells were collected using a cell scraper. For each condition, 10 μL was used to make a serial dilution and plated in duplicate on LB agar. Plates were incubated at 37°C overnight. For each condition, CFUs were counted and reported as CFUs/mL.

### Pyochelin and pyoverdine measurements

For each condition, BMDM were permeabilized with 0.1% Triton X-100 for 5 m and 100 μL of infected cell supernatant was transferred in duplicate into a black clear-bottom 96-well plate. The plate was read using a Varioskan Lux plate reader at Ex350/Em430nm (pyochelin) and Ex400/Em460nm (pyoverdine). For each sample, the medium fluorescence (background) was subtracted from the raw fluorescence values and the subtracted fluorescence values (RFU) were normalized on log_10_(CFU).

### Metal complexes with pyochelin and pyoverdine for BMDM treatments

Pyochelin I & II (Toronto Research Chemicals cat# P840365) and pyoverdine (Sigma cat# P8124) were purchased and used for the BMDM experiments. For pyochelin, 100 μM or 1 mM pyochelin was incubated with either 50 μM or 500 μM iron(III) chloride (Fe(III)) respectively, or 50 μM or 500 μM gallium(III) nitrate hydrate respectively, in 50mM Tris-HCl buffer pH 7.6 for 16h at room temperature. For pyoverdine, 100 μM or 1 mM pyoverdine was incubated with either 100 μM or 1 mM Fe(III) respectively, or 100 μM or 1 mM gallium(III) nitrate hydrate respectively, in 50mM Tris-HCl buffer pH 7.6 for 16h at room temperature. The solutions were then diluted 1:10 in macrophage growth medium, filtered sterilized and added to BMDM for 6 h.

### Pyochelin and pyoverdine fluorescence spectrum

For pyochelin spectra, 1 mM pyochelin was incubated with either 500 μM Fe(III) or 500 μM gallium(III) nitrate hydrate in 50mM Tris-HCl buffer pH 7.6 for 16h at room temperature. For pyoverdine spectrum, 1 mM pyoverdine was incubated with either 1 mM Fe(III) or 1 mM gallium(III) nitrate hydrate in 50mM Tris-HCl buffer pH 7.6 for 16h at room temperature. The solutions were then diluted 1:10 in macrophage growth medium and 100 μL of each solution was transferred in duplicate into a black clear-bottom 96-well plate. The plate was read using a Varioskan Lux plate reader at Ex350 for pyochelin and Ex400 for pyoverdine.

### Labile iron pool quantification

To quantify the labile iron pool in infected BMDM, we used a colorimetric method described by Abbasi et al. [33] with the following modifications. Briefly, BMDM were washed with 1X PBS and harvested in 5mM EDTA 1X EDTA. The supernatant was removed and cells were lysed in 200 μL Pierce RIPA buffer (ThermoFisher Scientific cat# 89900) with sonication for 20 min. After lysis, samples were centrifuged at 16,000 g for 10 min at 4°C and supernatants were transferred into clean tubes for further analysis. Iron standards were prepared from Fe(III) in 2% nitric acid, ranging from 0 to 500 μM. Samples (100 μL) and iron standards (100 μL) were transferred into different clean tubes. Ammonium acetate buffer (pH 4.5, 2.5 M) (100 μL) and labile iron working solution (5 mM ferene [Sigma cat# 82940] and 10 mM ascorbic acid [Sigma cat# 255564] prepared in ammonium acetate buffer pH 4.5, 2.5 M) (120 μL) were added to all tubes. The mixtures were then vortexed and incubated overnight at room temperature. The next day, the samples and standards were spun at 16,000 g for 5 m, and 200 μL were transferred into a 96 well plate for absorbance measurements. Absorbance was recorded at 595 nm using a Varioskan Lux plate reader. Iron concentrations were interpolated from the standard curve generated from the iron standards. These concentrations were normalized to the amount of protein analyzed (i.e., nmol of iron per mg of protein).

### Protein quantification

Protein content in cell lysates were measured using the BCA Total protein assay kit (ThermoFisher Scientific cat# 23227), following the manufacturer’s protocol. In brief, working solution was prepared using reagent A and B at a 50:1 ratio, respectively. BSA standards (10 μL) and cell lysates (10 μL) were added into a 96 well plate. Then, 200 μL of Bradford working solution were added to each well. This was kept at 37°C for 30 min, then cooled to room temperature for 5 min. Absorbance was read at 562 nm. Protein concentrations in cell lysates were interpolated using the standard curve generated.

### Growth curve in gallium

Overnight cultures were diluted to reach OD_600_~0.005. Ten microliters of these cultures were inoculated in 96-well plates in LB broth containing different concentrations of gallium (III) nitrate hydrate (Sigma cat# 289892). Fifty microliters of mineral oil was added in each well to prevent evaporation. Plates were incubated at 37°C for 20 h, shaking for 5 s every hour and absorbance at 600 nm was recorded using a Varioskan Lux microplate reader (Thermo Fisher Scientific, cat# NC1141718).

### BMDM treatment with gallium for BMDM toxicity

Gallium was added to uninfected BMDM to final concentrations of 500, 750, 1000 and 2000 μM for 6 h. Cytotoxicity was performed using a LDH cytotoxicity assay as described above.

### BMDM treatment with gallium during infection

BMDM were infected as described above. For cytotoxicity assays, gallium was added immediately after infection to final concentrations of 100, 200, 375, 500, and 750μM for 6 h. For CFU counts, gallium was added immediately after infection to a final concentration of 750 μM for 3 h, 6 h, or 24 h.

### RNA-seq library preparation

BMDM were infected with *P*. *aeruginosa* strains at MOI: 100 for 3h or 6h. Cells were washed and preserved in RNAlater (Invitrogen cat# AM7021) until RNA extraction. Total BMDM and bacterial RNA was extracted using a RNeasy Mini Kit (Qiagen, cat# 74106) and resuspended in 30 μL of elution buffer. Of this volume, 25 μL was used for bacterial RNA libraries and 5 μL was used for BMDM RNA libraries. For bacterial RNA libraries, RNA from host nucleic acids and bacterial rRNA was depleted using MicrobEnrich Kit (Invitrogen, cat# AM1901) and MicrobExpress Bacterial mRNA Enrichment Kit (Invitrogen, cat# AM1905) respectively, according to manufacturer’s instructions. RNA-seq libraries were prepared for bacteria using the KAPA RNA HyperPrep Kit (KAPA BioSystem, cat# KK8540) and BMDM using the KAPA mRNA HyperPrep Kit (KAPA BioSystem, cat# KK8580), according to manufacturer’s instruction. Libraries were quantified using Quant-iT dsDNA Assay Kit, high sensitivity (Invitrogen, cat# Q33120) and the quality was verified on an Agilent 4150 TapeStation System using D1000 High Sensitivity DNA Tape (Agilent, cat#5067–5584). Libraries were pooled to equimolar amounts and sequenced using a NextSeq 500 instrument to a depth of 400 million reads per sample (75 bp, single-ends) at the Cedars-Sinai Genomics Core Facility.

### RNA-seq data analyses

For clinical isolates, publicly available data was analyzed (Accession No. PRJEB24688) [39]. RNA sequencing data from WT and AzEvC10 PAO1 is available in the NCBI Sequence Read Archive (Bioproject Accession # PRJNA934930). RNA-seq data were analyzed using CLC Genomics workbench 20 (version 20.0.1). The *P*. *aeruginosa* PAO1 genome (Genbank accession #GCA_000006765.1) was as a reference for RNA sequencing read alignment. Differential Expression for RNA-Seq (version 2.2) analysis was performed to compare whole transcriptomes between the following groups: WT PAO1 vs AzEvC10 mutant at 3 hpi and 6 hpi, each strain during BMDM infection at 3 hpi and 6 hpi vs LB growth, and CF sputum vs *in vitro* clinical isolates. Reads were normalized in transcripts per million reads (TPM). Gene Set Test (version 1.1) using Gene Ontology (GO) biological processes annotations was performed to reveal enriched biological processes in the AzEvC10 mutant after 3 h and 6 h of BMDM infection. Venn Diagram for RNA-Seq tool (version 0.2) analysis was performed using a minimal absolute fold-change of 1.5 and false discovery rate (FDR) *p*-value of 0.05. The lists of DEGs are available in S1 and S2 Tables.

### Whole genome sequencing of clinical *P*. *aeruginosa* isolates

*P*. *aeruginosa* clinical isolates from CF (n = 11) and non-CF (n = 29) ICU patients were a generous gift from the Pulmonary Translational Research Core at University of Pittsburgh. These isolates were sent for whole genome sequencing to the Microbial Genome Sequencing Center (MiGs). Genome sequencing data is available in the NCBI Sequence Read Archive (Bioproject Accession # PRJNA934930). These samples were analyzed alongside publicly available whole genome sequencing data from CF sputum (n = 5) (Accession No. PRJEB24688) [39]. Variant calling program Breseq version 0.35.5 was utilized to identify mutations in the isolates’ genomes relative to the PAO1 reference (Genbank accession #GCA_000006765.1) [90]. The identified mutations were filtered for indels and non-synonymous SNPs and then subsequently filtered to identify unique mutations found only in the *rne* gene.

### Statistical analyses

Normality and homogeneity of variance were assessed by Shapiro-Wilk and Brown-Forsythe tests, respectively. Data was log-transformed prior to analysis where necessary to meet assumptions necessary for parametric testing, else non-parametric rank testing was used. Based on data distributions, analysis was performed for matched data with paired Student t-test or Wilcoxon matched pairs rank test; for two groups with a two-sample Student t-test or the nonparametric Mann-Whitney; for three or more groups with ANOVA followed by Tukey’s test or the non-parametric Kruskal-Wallis test with Dunn’s correction. Where data were collected across experimental repeats or repeated measures, data were considered as matched sets, and RM-ANOVA was used. Cytotoxicity dose curves were analyzed with two-way ANOVA. For all parametric testing, residuals were inspected to confirm fit. Fisher’s exact test was used to analyze categorical data. All testing was considered significant at the two-tailed p-value of <0.05. Analysis performed with GraphPad Prism v9. The *p*-values are listed in S5 Table.

## Supporting information

S1 Fig Susceptibility to alveolar macrophages, BMDM growth medium and amikacin. **A.** Neutrophil and AEC cytotoxicity assessed by LDH assay at 6 hpi by WT or AzEVC10. **B.** BMDM positivity for the alveolar macrophage markers CD64 and Siglec F by flow cytometry. **C.** Alveolar macrophages were infected with WT PAO1 or the AzEvC10 mutant MOI:100 for 6 h. Dotted lines represent the initial infection inoculum, 2.5 x 10^7^ CFU/mL. Bacterial burden at 6 hpi was determined by viable CFU plate counts. **D.** Bacterial survival determined by viable CFU plate counts after inoculating BMDM growth medium with *P*. *aeruginosa* strains in the absence of BMDM. Dotted line represents the initial inoculum, 2.5 x 10^7^ CFU/mL. **E.** BMDM growth medium was inoculated with WT PAO1 and AzEvC10 mutant with or without amikacin 50, 100 or 200 μg/mL and incubated for 60 min at 37°C and 5% CO_2_. Dotted line represents the initial inoculum, 2.5 x 10^7^ CFU/mL. No CFU was recovered at 200 μg/mL for both WT PAO1 and AzEvC10 mutant, and this concentration was used for assays to kill extracellular bacteria during BMDM infection. n = 3–6 independent replicates for each experiment. **p<0.01. See S5 Table for statistical tests used and exact *p*-values.
(PDF)

S2 Fig Virulence of the AzEvC10 mutant is not reversed by *nalD* complementation or *mexAB* efflux pump deletion. **A.** Genome diagram showing coverage of sequencing reads aligning to the *nalD* gene in WT PAO1 and the AzEvC10 mutant. The single base substitution leading to *nalD*_T158P_ in AzEvC10 mutant is highlighted in red. **B-C.** *nalD* complementation in AzEvC10 mutant was confirmed by RT-qPCR **(B)** and aztreonam MIC. **(C). D-E.** BMDM were infected with either WT PAO1, AzEvC10, or AzEvC10 carrying pMQ72::*nalD*_WT_ at MOI:100 for 6h. **D.** Bacterial burden determined by viable CFU plate counts. Dotted line represents the initial infection inoculum, 2.5 x 10^7^ CFU/mL. **E.** BMDM cytotoxicity was assessed by LDH assay. **F.** Gel electrophoresis showing *mexAB* deletion in WT PAO1 and AzEvC10 strains. **G.** Aztreonam MIC by Etest. **H-I.** BMDM were infected with either WT PAO1, AzEvC10 or the strains containing the *mexAB* deletion at MOI:100 for 6h. **H.** Bacterial burden determined by viable CFU plate counts. Dotted line represents the initial infection inoculum, 2.5 x 10^7^ CFU/mL. **I.** BMDM cytotoxicity was assessed by LDH assay. n = 3–4 independent replicates for each experiment. *p<0.05, **p<0.01, and ***p<0.001. See S5 Table for statistical tests used and exact *p*-values.
(PDF)

S3 Fig Pyochelin and pyoverdine fluorescence in macrophage growth medium. **A-B.** Fluorescence spectra of 100 μM pyochelin incubated with either 50μM Fe(III) or 50μM gallium with an excitation at 350nm. **C-D.** Fluorescence spectra of 100 μM pyoverdine incubated with either 100μM Fe(III) or 100μM gallium with an excitation at 400nm. **E.** Fluorescence values (RFU) after medium background subtraction of pyochelin (Ex350/Em430) and pyoverdine (Ex400/Em460) at 3 hpi and 6 hpi. **F.** Pyochelin and pyoverdine production by given strains measured by fluorescence (Ex350/Em430 for pyochelin and Ex400/Em460 for pyoverdine) and normalized to log_10_(CFU) after 6h growth in macrophage growth media without macrophages. See S5 Table for statistical tests used and exact *p*-values.
(PDF)

S4 Fig Ferric-pyochelin is sufficient to induce macrophage ferroptosis. **A-B.** BMDM were infected with WT PAO1 or the AzEvC10 mutant, MOI:100 for 3 h or 6 h. **A.** BMDM cell death was measured by flow cytometry and characterized by annexin V and PI mean fluorescence intensity (MFI). **B.** The labile iron pool was quantified by colorimetry at 6 hpi. **C-E.** Uninfected BMDM were treated with 100 μM of either Fe(III), pyoverdine (Pvd), ferric-pyoverdine (Pvd_Fe(III)_), pyochelin (Pch), or ferric-pyochelin (Pch_Fe(III)_) for 6h. Quantification of ROS presented in mean fluorescence intensity (MFI) **(C,E).** Quantification of lipid peroxidation in BMDM by flow cytometry presented as the reciprocal of the ratio of red (Ex561/Em582)/green (Ex488/Em525) fluorescence intensities **(D).** n = 3 independent replicates for each experiment. *p<0.05, **p<0.01, ***p<0.001, ****p<0.0001. See S5 Table for statistical tests used and exact *p*-values.
(PDF)

S5 Fig Gallium treatment decreases pyochelin-induced ferroptosis in macrophages. **A.** Uninfected BMDM were treated with increasing concentrations of gallium for 6 h. BMDM cytotoxicity assessed by LDH assay at 6 h post-treatment. **B.** Fluorescence values (RFU) after medium background subtraction of pyochelin (Ex350/Em430) and pyoverdine (Ex400/Em460) at 3 hpi, 6 hpi (MOI:100) and 24 hpi (MOI:1) with or without gallium. **C-F.** BMDM were infected with either WT PAO1 or the AzEvC10 mutant (MOI:100) and treated with 750 μM gallium for 3 h **(C-D)** and 6 h **(E-F)**. Quantification of ROS and lipid peroxidation were performed by flow cytometry. **G-H.** BMDM were infected with either WT PAO1 or the AzEvC10 mutant (MOI:1) and treated with 750 μM gallium for 24 h. Quantification of ROS and lipid peroxidation were performed by flow cytometry. **I-K.** Uninfected BMDM were treated with 10 μM or 100 μM of either gallium, pyochelin (Pch), ferric-pyochelin (Pch_Fe(III)_) or gallium-pyochelin (Pch_Ga(III)_) for 6h. Quantification of ROS **(I-J)** and lipid peroxidation **(K)** were performed by flow cytometry. n = 3 independent replicates for each experiment. *p<0.05, **p<0.01, ***p<0.001, ****p<0.0001. See S5 Table for statistical tests used and exact *p*-values.
(PDF)

S1 Table 50 most upregulated genes in AzEvC10 during macrophage infection. (XLSX)

S2 Table Common DEGs between sputum and AzEvC10 during macrophage infection. (XLSX)

S3 Table List of strains. (XLSX)

S4 Table List of primers. (XLSX)

S5 Table Statistics. (DOCX)
