# Supplementary material for: A compensatory RNase E variation increases Iron Piracy and Virulence in multidrug-resistant Pseudomonas aeruginosa during Macrophage infection
Source: PLoS Pathog. 2023 Apr 7;19(4):e1010942. doi: 10.1371/journal.ppat.1010942 (PMC10115287; doi:10.1371/journal.ppat.1010942)
Supplement: S1 Fig — A. Neutrophil and AEC cytotoxicity assessed by LDH assay at 6 hpi by WT or AzEVC10. B. BMDM positivity for the alveolar macrophage markers CD64 and Siglec F by flow cytometry. C. Alveolar macrophages were infected with WT PAO1 or the AzEvC10 mutant MOI:100 for 6 h. Dotted lines represent the initial infection inoculum, 2.5 x 10^7^ CFU/mL. Bacterial burden at 6 hpi was determined by viable CFU plate counts. D. Bacterial survival determined by viable CFU plate counts after inoculating BMDM growth medium with P. aeruginosa strains in the absence of BMDM. Dotted line represents the initial inoculum, 2.5 x 10^7^ CFU/mL. E. BMDM growth medium was inoculated with WT PAO1 and AzEvC10 mutant with or without amikacin 50, 100 or 200 μg/mL and incubated for 60 min at 37°C and 5% CO2. Dotted line represents the initial inoculum, 2.5 x 10^7^ CFU/mL. No CFU was recovered at 200 μg/mL for both WT PAO1 and AzEvC10 mutant, and this concentration was used for assays to kill extracellular bacteria during BMDM infection. n = 3–6 independent replicates for each experiment. **p<0.01. See S5 Table for statistical tests used and exact p-values. (PDF) [file ppat.1010942.s001.pdf]

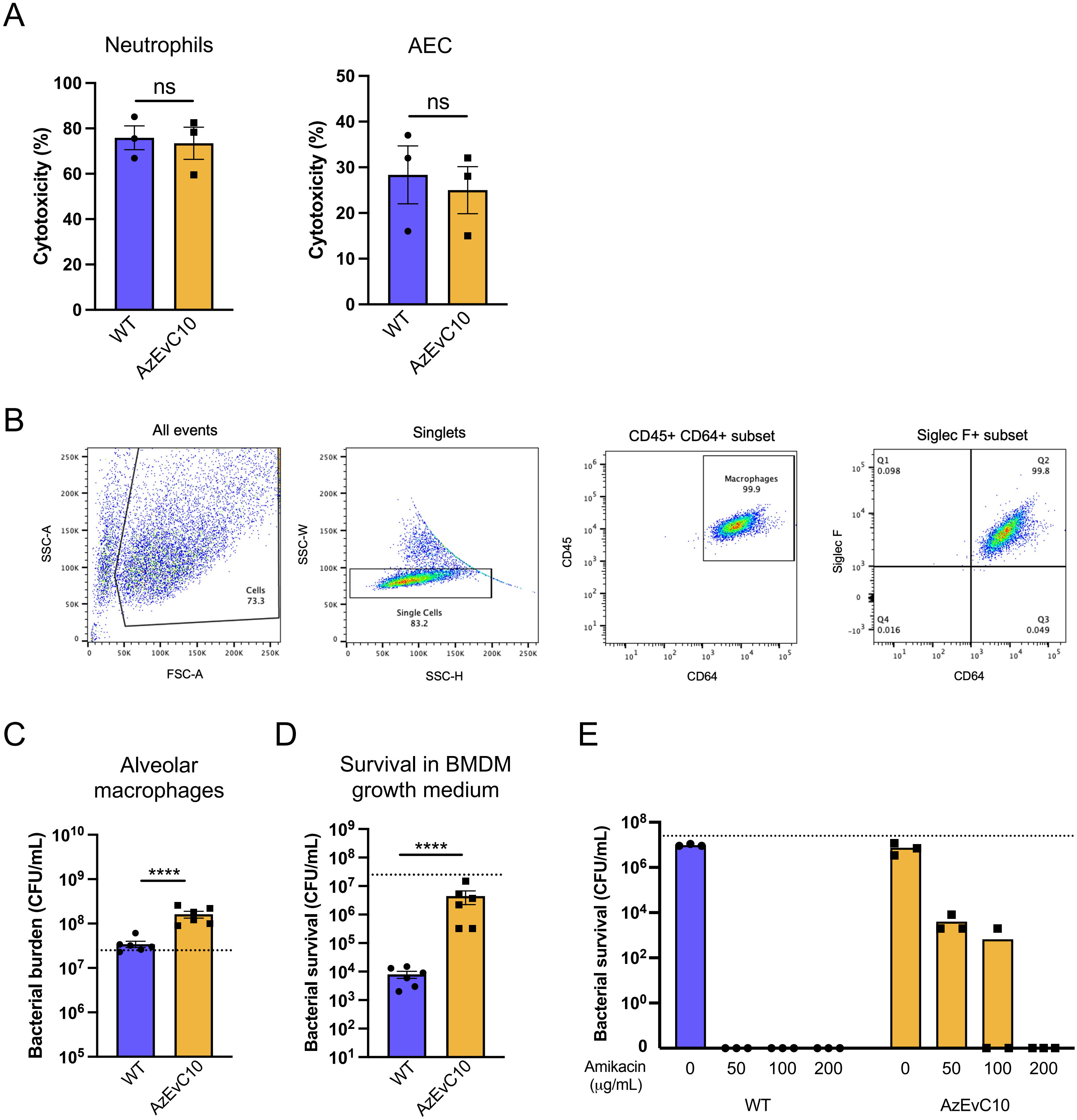

Figure S1. Susceptibility to alveolar macrophages, BMDM growth medium and amikacin

A. Neutrophil and AEC cytotoxicity assessed by LDH assay at 6 hpi by WT or AzEVC10. **B.** BMDM positivity for the alveolar macrophage markers CD64 and Siglec F by flow cytometry. **C.** Alveolar macrophages were infected with WT PAO1 or the AzEvC10 mutant MOI:100 for 6 h. Dotted lines represent the initial infection inoculum, 2.5 x 10^7 CFU/mL. Bacterial burden at 6 hpi was determined by viable CFU plate counts. **D.** Bacterial survival determined by viable CFU plate counts after inoculating BMDM growth medium with *P. aeruginosa* strains in the absence of BMDM. Dotted line represents the initial inoculum, 2.5 x 10^7 CFU/mL. **E.** BMDM growth medium was inoculated with WT PAO1 and AzEvC10 mutant with or without amikacin 50, 100 or 200 μg/mL and incubated for 60 min at 37°C and 5% CO_2. Dotted line represents the initial inoculum, 2.5 x 10^7 CFU/mL. No CFU was recovered at 200 μg/mL for both WT PAO1 and AzEvC10 mutant, and this concentration was used for assays to kill extracellular bacteria during BMDM infection. n=3-6 independent replicates for each experiment. ***p*<0.01. See Table S5 for statistical tests used and exact *p*-values.
