# Supplementary material for: A compensatory RNase E variation increases Iron Piracy and Virulence in multidrug-resistant Pseudomonas aeruginosa during Macrophage infection
Source: PLoS Pathog. 2023 Apr 7;19(4):e1010942. doi: 10.1371/journal.ppat.1010942 (PMC10115287; doi:10.1371/journal.ppat.1010942)
Supplement: S2 Fig — A. Genome diagram showing coverage of sequencing reads aligning to the nalD gene in WT PAO1 and the AzEvC10 mutant. The single base substitution leading to nalDT158P in AzEvC10 mutant is highlighted in red. B-C. nalD complementation in AzEvC10 mutant was confirmed by RT-qPCR (B) and aztreonam MIC. (C). D-E. BMDM were infected with either WT PAO1, AzEvC10, or AzEvC10 carrying pMQ72::nalDWT at MOI:100 for 6h. D. Bacterial burden determined by viable CFU plate counts. Dotted line represents the initial infection inoculum, 2.5 x 10^7^ CFU/mL. E. BMDM cytotoxicity was assessed by LDH assay. F. Gel electrophoresis showing mexAB deletion in WT PAO1 and AzEvC10 strains. G. Aztreonam MIC by Etest. H-I. BMDM were infected with either WT PAO1, AzEvC10 or the strains containing the mexAB deletion at MOI:100 for 6h. H. Bacterial burden determined by viable CFU plate counts. Dotted line represents the initial infection inoculum, 2.5 x 10^7^ CFU/mL. I. BMDM cytotoxicity was assessed by LDH assay. n = 3–4 independent replicates for each experiment. *p<0.05, **p<0.01, and ***p<0.001. See S5 Table for statistical tests used and exact p-values. (PDF) [file ppat.1010942.s002.pdf]

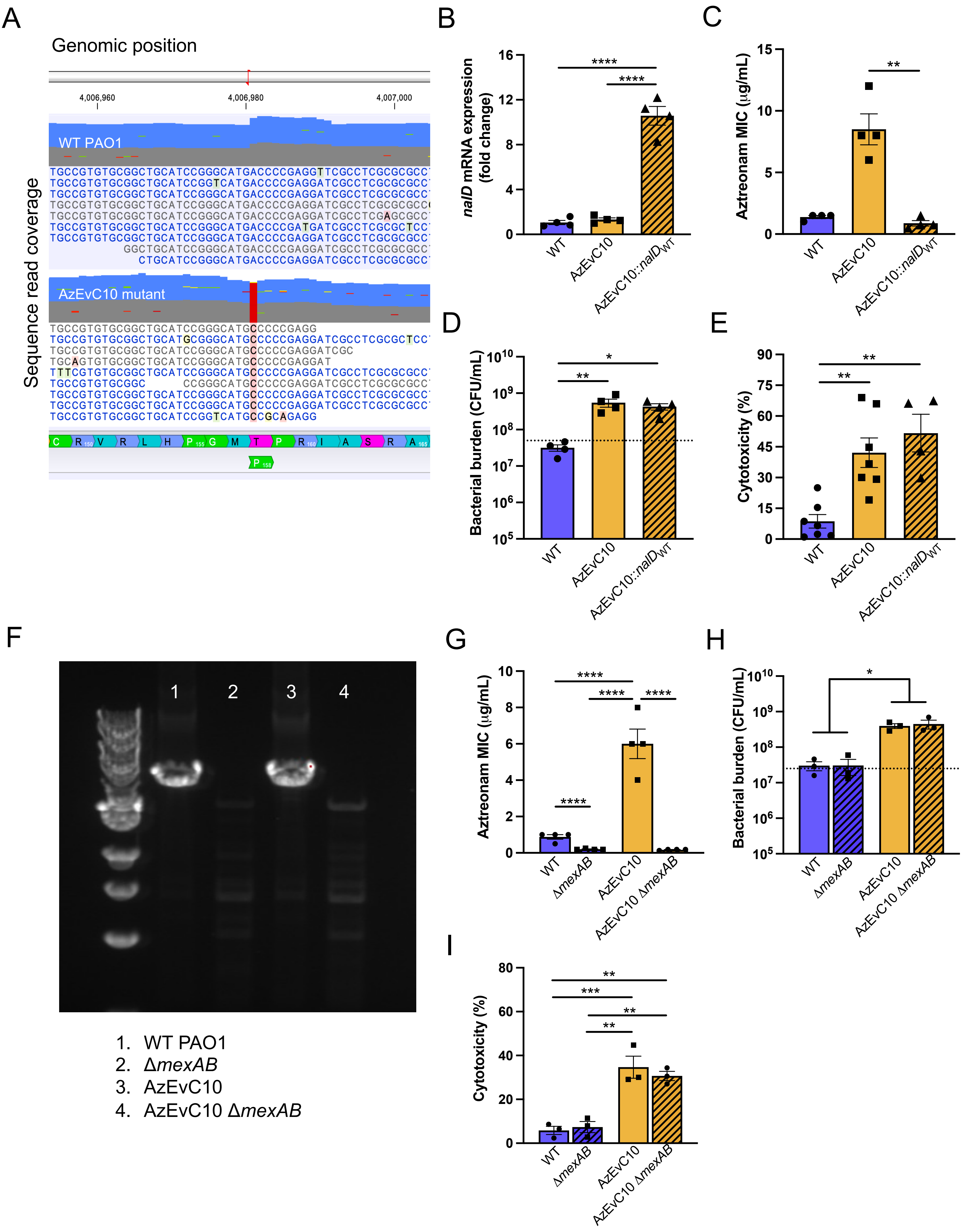

A
Genomic position
4,006,960
4,006,980
4,007,000
Sequence read coverage
WT PAO1
AzEvC10 mutant
B
nalD mRNA expression (fold change)
WT
AzEvC10
AzEvC10::nalDWT
C
Aztreonam MIC (μg/mL)
D
Bacterial burden (CFU/mL)
E
Cytotoxicity (%)
F
1
2
3
4
1. WT PAO1
2. ΔmexAB
3. AzEvC10
4. AzEvC10 ΔmexAB
G
Aztreonam MIC (μg/mL)
WT
ΔmexAB
AzEvC10
AzEvC10 ΔmexAB
H
Bacterial burden (CFU/mL)
I
Cytotoxicity (%)

Figure S2. Virulence of the AzEvC10 mutant is not reversed by *nalD* complementation or *mexAB* efflux pump deletion

A. Genome diagram showing coverage of sequencing reads aligning to the *nalD* gene in WT PAO1 and the AzEvC10 mutant. The single base substitution leading to $nalD_{T158P}$ in AzEvC10 mutant is highlighted in red. **B-C.** *nalD* complementation in AzEvC10 mutant was confirmed by RT-qPCR **(B)** and aztreonam MIC **(C).** **D-E.** BMDM were infected with either WT PAO1, AzEvC10, or AzEvC10 carrying pMQ72::$nalD_{WT}$ at MOI:100 for 6h. **D.** Bacterial burden determined by viable CFU plate counts. Dotted line represents the initial infection inoculum, 2.5 x 10^7 CFU/mL. **E.** BMDM cytotoxicity was assessed by LDH assay. **F.** Gel electrophoresis showing *mexAB* deletion in WT PAO1 and AzEvC10 strains. **G.** Aztreonam MIC was by Etest strip. **H-I.** BMDM were infected with either WT PAO1, AzEvC10 or the strains containing the *mexAB* deletion at MOI:100 for 6h. **H.** Bacterial burden determined by viable CFU plate counts. Dotted line represents the initial infection inoculum, 2.5 x 10^7 CFU/mL. **I.** BMDM cytotoxicity was assessed by LDH assay. n=3-4 independent replicates for each experiment. *$p<0.05$, **$p<0.01$, and ***$p<0.001$. See Table S5 for statistical tests used and exact *p*-values.
