# Supplementary material for: A compensatory RNase E variation increases Iron Piracy and Virulence in multidrug-resistant Pseudomonas aeruginosa during Macrophage infection
Source: PLoS Pathog. 2023 Apr 7;19(4):e1010942. doi: 10.1371/journal.ppat.1010942 (PMC10115287; doi:10.1371/journal.ppat.1010942)
Supplement: S3 Fig — A-B. Fluorescence spectra of 100 μM pyochelin incubated with either 50μM Fe(III) or 50μM gallium with an excitation at 350nm. C-D. Fluorescence spectra of 100 μM pyoverdine incubated with either 100μM Fe(III) or 100μM gallium with an excitation at 400nm. E. Fluorescence values (RFU) after medium background subtraction of pyochelin (Ex350/Em430) and pyoverdine (Ex400/Em460) at 3 hpi and 6 hpi. F. Pyochelin and pyoverdine production by given strains measured by fluorescence (Ex350/Em430 for pyochelin and Ex400/Em460 for pyoverdine) and normalized to log10(CFU) after 6h growth in macrophage growth media without macrophages. See S5 Table for statistical tests used and exact p-values. (PDF) [file ppat.1010942.s003.pdf]

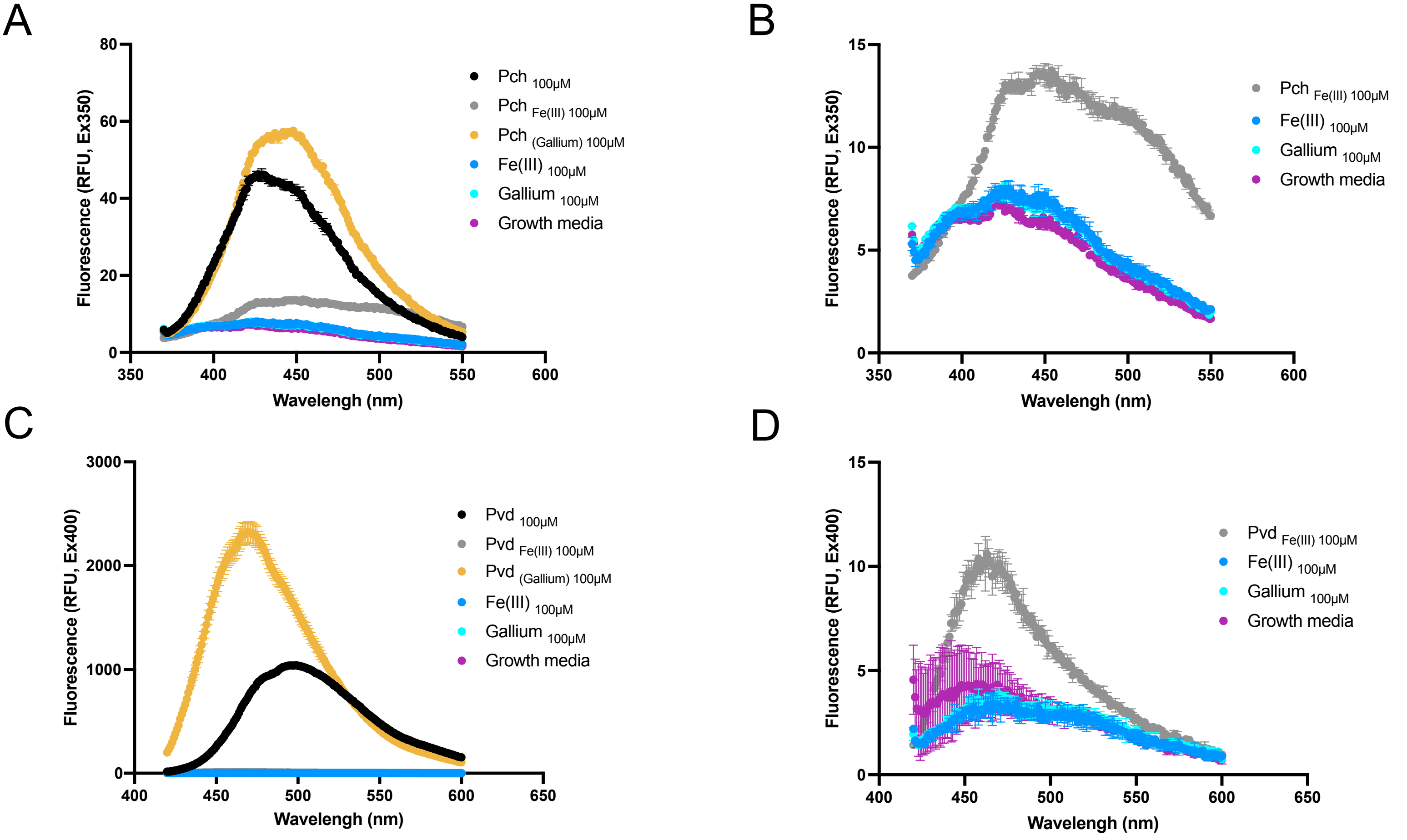

E

Pyochelin $_{Ex350/Em430}$				
	3 hpi		6 hpi	
Bacterial strain	Fluorescence	SD	Fluorescence	SD
WT	1.083	1.313	3.908	5.691
AzEvC10	5.986	1.892	70.70	15.07
$nalD_{T158P}$	0.134	0.207	0.190	0.194
$rne_{\Delta 50bp}$	0.856	0.111	55.54	3.575
rne:Tn	10.29	3.237	69.91	5.086
AzEvC10$_{rneWT}$	0.322	0.109	16.11	1.025

Pyoverdine $_{Ex400/Em460}$				
	3 hpi		6 hpi	
Bacterial strain	Fluorescence	SD	Fluorescence	SD
WT	0.932	0.988	30.75	52.45
AzEvC10	45.12	15.44	645.1	100.7
$nalD_{T158P}$	0.480	0.248	1.021	0.702
$rne_{\Delta 50bp}$	5.260	0.790	242.9	17.92
rne:Tn	111.1	32.02	705.5	67.20
AzEvC10$_{rneWT}$	2.395	0.126	51.48	6.442

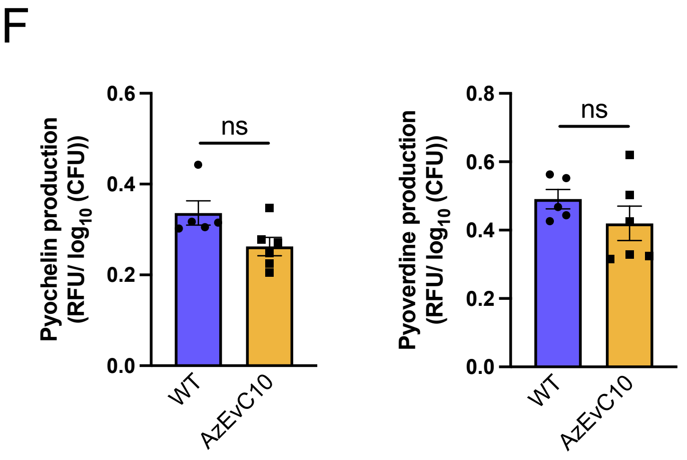

Figure S3. Pyochelin and pyoverdine fluorescence in macrophage growth medium

A-B. Fluorescence spectra of 100 µM pyochelin incubated with either 50µM Fe(III) or 50µM gallium with an excitation at 350nm. **C-D.** Fluorescence spectra of 100 µM pyoverdine incubated with either 100µM Fe(III) or 100µM gallium with an excitation at 400nm. **E.** Fluorescence values (RFU) after medium background subtraction of pyochelin (Ex350/Em430) and pyoverdine (Ex400/Em460) at 3 hpi and 6 hpi. **F.** Pyochelin and pyoverdine production by given strains measured by fluorescence (Ex350/Em430 for pyochelin and Ex400/Em460 for pyoverdine) and normalized to log_{10}(CFU) after 6h growth in macrophage growth media without macrophages. See Table S5 for statistical tests used and exact *p*-values.
