# Supplementary material for: A compensatory RNase E variation increases Iron Piracy and Virulence in multidrug-resistant Pseudomonas aeruginosa during Macrophage infection
Source: PLoS Pathog. 2023 Apr 7;19(4):e1010942. doi: 10.1371/journal.ppat.1010942 (PMC10115287; doi:10.1371/journal.ppat.1010942)
Supplement: S4 Fig — A-B. BMDM were infected with WT PAO1 or the AzEvC10 mutant, MOI:100 for 3 h or 6 h. A. BMDM cell death was measured by flow cytometry and characterized by annexin V and PI mean fluorescence intensity (MFI). B. The labile iron pool was quantified by colorimetry at 6 hpi. C-E. Uninfected BMDM were treated with 100 μM of either Fe(III), pyoverdine (Pvd), ferric-pyoverdine (PvdFe(III)), pyochelin (Pch), or ferric-pyochelin (PchFe(III)) for 6h. Quantification of ROS presented in mean fluorescence intensity (MFI) (C,E). Quantification of lipid peroxidation in BMDM by flow cytometry presented as the reciprocal of the ratio of red (Ex561/Em582)/green (Ex488/Em525) fluorescence intensities (D). n = 3 independent replicates for each experiment. *p<0.05, **p<0.01, ***p<0.001, ****p<0.0001. See S5 Table for statistical tests used and exact p-values. (PDF) [file ppat.1010942.s004.pdf]

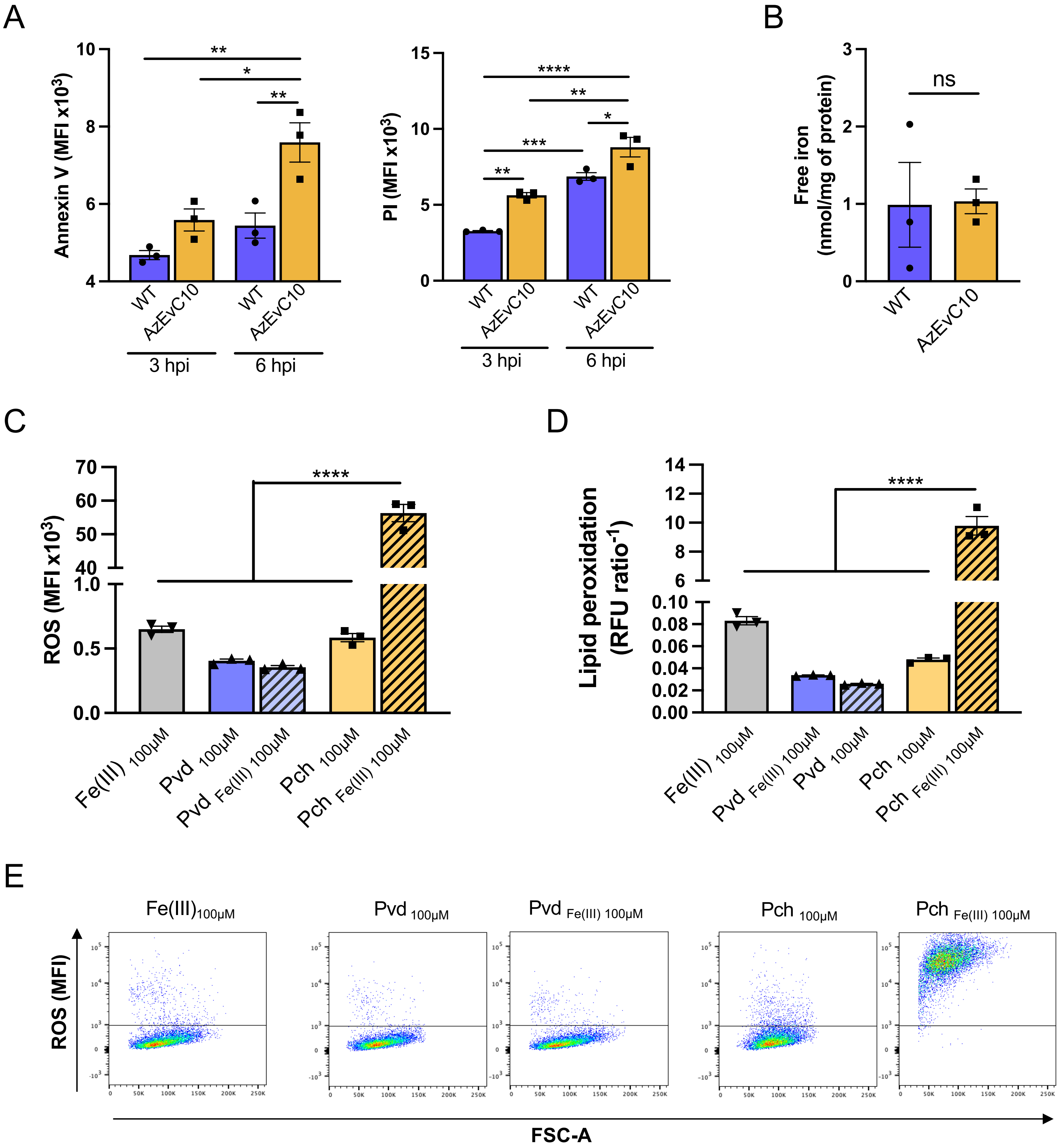

Figure S4. Ferric-pyochelin is sufficient to induce macrophage ferroptosis

A-B. BMDM were infected with WT PAO1 or the AzEvC10 mutant, MOI:100 for 3 h or 6 h. **A.** BMDM cell death was measured by flow cytometry and characterized by annexin V and PI mean fluorescence intensity (MFI). **B.** The labile iron pool was quantified by colorimetry at 6 hpi. **C-E.** Uninfected BMDM were treated with 100 µM of either Fe(III), pyoverdine (Pvd), ferric-pyoverdine ($Pvd_{Fe(III)}$), pyochelin (Pch), or ferric-pyochelin ($Pch_{Fe(III)}$) for 6h. Quantification of ROS presented in mean fluorescence intensity (MFI) **(C,E).** Quantification of lipid peroxidation in BMDM by flow cytometry presented as the reciprocal of the ratio of red (Ex561/Em582)/green (Ex488/Em525) fluorescence intensities **(D).** n=3 independent replicates for each experiment. *p<0.05, **p<0.01, ***p<0.001, ****p<0.0001. See Table S5 for statistical tests used and exact p-values.
