# Supplementary material for: A compensatory RNase E variation increases Iron Piracy and Virulence in multidrug-resistant Pseudomonas aeruginosa during Macrophage infection
Source: PLoS Pathog. 2023 Apr 7;19(4):e1010942. doi: 10.1371/journal.ppat.1010942 (PMC10115287; doi:10.1371/journal.ppat.1010942)
Supplement: S5 Fig — A. Uninfected BMDM were treated with increasing concentrations of gallium for 6 h. BMDM cytotoxicity assessed by LDH assay at 6 h post-treatment. B. Fluorescence values (RFU) after medium background subtraction of pyochelin (Ex350/Em430) and pyoverdine (Ex400/Em460) at 3 hpi, 6 hpi (MOI:100) and 24 hpi (MOI:1) with or without gallium. C-F. BMDM were infected with either WT PAO1 or the AzEvC10 mutant (MOI:100) and treated with 750 μM gallium for 3 h (C-D) and 6 h (E-F). Quantification of ROS and lipid peroxidation were performed by flow cytometry. G-H. BMDM were infected with either WT PAO1 or the AzEvC10 mutant (MOI:1) and treated with 750 μM gallium for 24 h. Quantification of ROS and lipid peroxidation were performed by flow cytometry. I-K. Uninfected BMDM were treated with 10 μM or 100 μM of either gallium, pyochelin (Pch), ferric-pyochelin (PchFe(III)) or gallium-pyochelin (PchGa(III)) for 6h. Quantification of ROS (I-J) and lipid peroxidation (K) were performed by flow cytometry. n = 3 independent replicates for each experiment. *p<0.05, **p<0.01, ***p<0.001, ****p<0.0001. See S5 Table for statistical tests used and exact p-values. (PDF) [file ppat.1010942.s005.pdf]

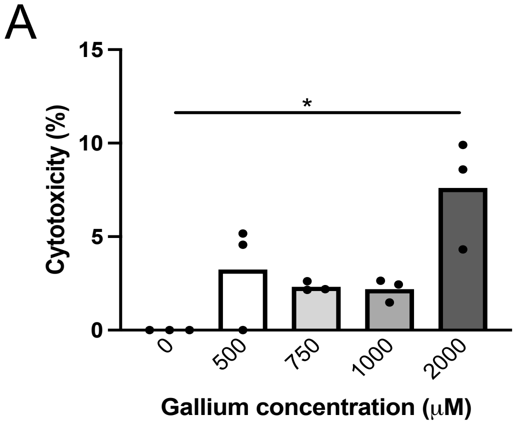

B

Pyochelin Ex350/ Em430						
	3 hpi		6 hpi		24 hpi	
Bacterial strain	Fluorescence	SD	Fluorescence	SD	Fluorescence	SD
WT	1.044	0.564	3.578	1.052	6.021	3.807
WT+gallium	0.908	0.591	1.857	0.910	0	0
AzEvC10	3.982	1.587	58.57	18.76	21.70	6.382
AzEvC10+gallium	1.452	0.488	9.886	4.103	0.917	1.087

Pyoverdine Ex3400 Em460						
	3 hpi		6 hpi		24 hpi	
Bacterial strain	Fluorescence	SD	Fluorescence	SD	Fluorescence	SD
WT	0.683	0.938	21.31	14.20	124.0	32.44
WT+gallium	0.493	0.627	2.191	2.626	0	0
AzEvC10	32.68	16.62	543.3	141.8	267.9	32.51
AzEvC10+gallium	6.668	1.509	74.01	30.73	24.78	17.31

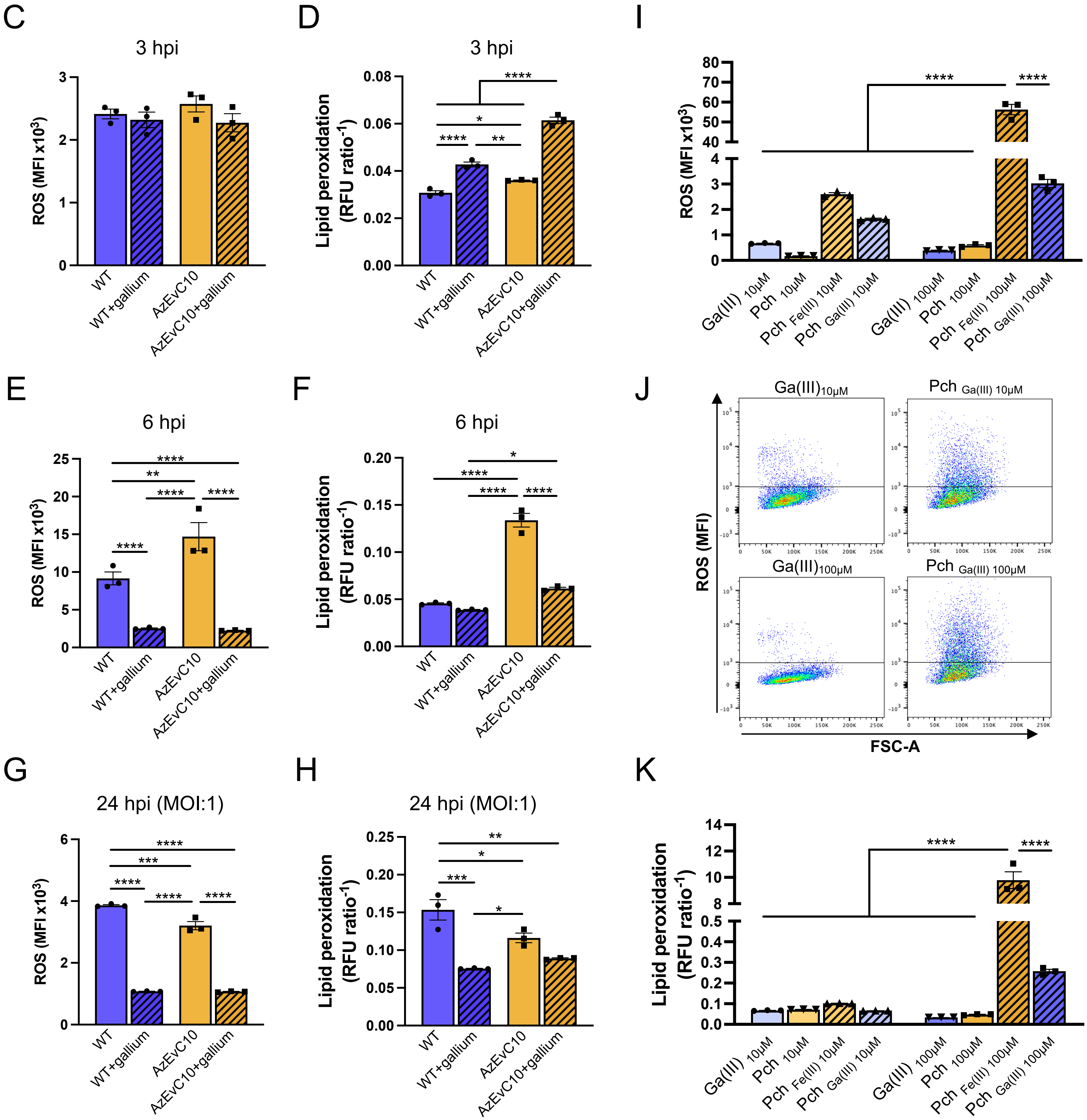

Figure S5. Gallium treatment decreases pyochelin-induced ferroptosis in macrophages

A. Uninfected BMDM were treated with increasing concentrations of gallium for 6 h. BMDM cytotoxicity assessed by LDH assay at 6 h post-treatment. **B.** Fluorescence values (RFU) after medium background subtraction of pyochelin (Ex350/Em430) and pyoverdine (Ex400/Em460) at 3 hpi, 6 hpi (MOI:100) and 24 hpi (MOI:1) with or without gallium. **C-F.** BMDM were infected with either WT PAO1 or the AzEvC10 mutant (MOI:100) and treated with 750 μM gallium for 3 h **(C-D)** and 6 h **(E-F)**. Quantification of ROS and lipid peroxidation were performed by flow cytometry. **G-H.** BMDM were infected with either WT PAO1 or the AzEvC10 mutant (MOI:1) and treated with 750 μM gallium for 24 h. Quantification of ROS and lipid peroxidation were performed by flow cytometry. **I-K.** Uninfected BMDM were treated with 10 μM or 100 μM of either gallium, pyochelin (Pch), ferric-pyochelin ($Pch_{Fe(III)}$) or gallium-pyochelin ($Pch_{Ga(III)}$) for 6h. Quantification of ROS **(I-J)** and lipid peroxidation **(K)** were performed by flow cytometry. n=3 independent replicates for each experiment. *$p<0.05$, **$p<0.01$, ***$p<0.001$, ****$p<0.0001$. See Table S5 for statistical tests used and exact *p*-values.
