# Supplementary material for: A compensatory RNase E variation increases Iron Piracy and Virulence in multidrug-resistant Pseudomonas aeruginosa during Macrophage infection
Source: PLoS Pathog. 2023 Apr 7;19(4):e1010942. doi: 10.1371/journal.ppat.1010942 (PMC10115287; doi:10.1371/journal.ppat.1010942)
Supplement: S5 Table — (DOCX) [file ppat.1010942.s010.docx]

Table S5: Statistics

| **Figure** | **Statistical Test** | **Comparison** | ***p*-value** |
| --- | --- | --- | --- |
| Fig. 1A  BMDM | Wilcoxon rank test  (paired, two-tailed) | WT vs. AzEvC10 | 0.0078 |
| Fig.1A  Neutrophils | T-test (unpaired, two-tailed) | WT vs. AzEvC10 | 0.1982 |
| Fig.1A  AEC | Mann-Whitney test | WT vs. AzEvC10 | 0.2000 |
| Fig. 1B | T-test (paired, two-tailed) | WT vs. AzEvC10 | 0.0226 |
| Fig. 1C | Mann-Whitney test | WT vs. AzEvC10 | 0.0286 |
| Fig. 1D | T-test (unpaired, two-tailed) | WT vs. AzEvC10 | 0.0003 |
| Fig. 2C | Kruskal-Wallis test | WT vs. AzEvC10 | 0.0013 |
|  |  | WT vs. *nalD*_T158P_ | 0.0095 |
|  |  | WT vs. *rne*_50bp_ | >0.9999 |
|  |  | WT vs. *rne*::tn | >0.9999 |
|  |  | WT vs. AzEvC10 *rne*_WT_ | 0.1516 |
|  |  | AzEvC10 vs. *nalD*_T158P_ | >0.9999 |
|  |  | AzEvC10 vs. *rne*_50bp_ | 0.0438 |
|  |  | AzEvC10 vs. *rne*::tn | 0.0183 |
|  |  | AzEvC10 vs. AzEvC10 *rne*_WT_ | >0.9999 |
|  |  | *nalD*_T158P_ vs. *rne*_50bp_ | 0.2073 |
|  |  | *nalD*_T158P_ vs. *rne*::tn | 0.0982 |
|  |  | *nalD*_T158P_ vs. AzEvC10 *rne*_WT_ | >0.9999 |
|  |  | *rne*_50bp_ vs. *rne*::tn | >0.9999 |
|  |  | *rne*_50bp_ vs. AzEvC10 *rne*_WT_ | >0.9999 |
|  |  | *rne*::tn vs. AzEvC10 *rne*_WT_ | 0.9141 |
| Fig. 2D | ANOVA (One-way) | WT vs. AzEvC10 | <0.0001 |
|  |  | WT vs. *nalD*_T158P_ | 0.1874 |
|  |  | WT vs. *rne*_50bp_ | <0.0001 |
|  |  | WT vs. *rne*::tn | <0.0001 |
|  |  | WT vs. AzEvC10 *rne*_WT_ | <0.0001 |
|  |  | AzEvC10 vs. *nalD*_T158P_ | 0.0002 |
|  |  | AzEvC10 vs. *rne*_50bp_ | 0.0939 |
|  |  | AzEvC10 vs. *rne*::tn | 0.0078 |
|  |  | AzEvC10 vs. AzEvC10 *rne*_WT_ | 0.5716 |
|  |  | *nalD*_T158P_ vs. *rne*_50bp_ | <0.0001 |
|  |  | *nalD*_T158P_ vs. *rne*::tn | <0.0001 |
|  |  | *nalD*_T158P_ vs. AzEvC10 *rne*_WT_ | 0.0527 |
|  |  | *rne*_50bp_ vs. *rne*::tn | 0.9765 |
|  |  | *rne*_50bp_ vs. AzEvC10 *rne*_WT_ | 0.0029 |
|  |  | *rne*::tn vs. AzEvC10 *rne*_WT_ | 0.0001 |
| Fig. 2E | Kruskal-Wallis test | WT vs. AzEvC10 | 0.1571 |
|  |  | WT vs. *nalD*_T158P_ | >0.9999 |
|  |  | WT vs. *rne*_50bp_ | 0.0012 |
|  |  | WT vs. *rne*::tn | <0.0001 |
|  |  | WT vs. AzEvC10 *rne*_WT_ | >0.9999 |
|  |  | AzEvC10 vs. *nalD*_T158P_ | 0.8591 |
|  |  | AzEvC10 vs. *rne*_50bp_ | >0.9999 |
|  |  | AzEvC10 vs. *rne*::tn | 0.5207 |
|  |  | AzEvC10 vs. AzEvC10 *rne*_WT_ | >0.9999 |
|  |  | *nalD*_T158P_ vs. *rne*_50bp_ | 0.0152 |
|  |  | *nalD*_T158P_ vs. *rne*::tn | 0.0012 |
|  |  | *nalD*_T158P_ vs. AzEvC10 *rne*_WT_ | >0.9999 |
|  |  | *rne*_50bp_ vs. *rne*::tn | >0.9999 |
|  |  | *rne*_50bp_ vs. AzEvC10 *rne*_WT_ | 0.2076 |
|  |  | *rne*::tn vs. AzEvC10 *rne*_WT_ | 0.0377 |
| Fig. 2F | Kruskal-Wallis test | WT vs. AzEvC10 | 0.0265 |
|  |  | WT vs. *nalD*_T158P_ | >0.9999 |
|  |  | WT vs. *rne*_50bp_ | 0.1554 |
|  |  | WT vs. *rne*::tn | 0.0019 |
|  |  | WT vs. AzEvC10 *rne*_WT_ | >0.9999 |
|  |  | AzEvC10 vs. *nalD*_T158P_ | 0.2137 |
|  |  | AzEvC10 vs. *rne*_50bp_ | >0.9999 |
|  |  | AzEvC10 vs. *rne*::tn | >0.9999 |
|  |  | AzEvC10 vs. AzEvC10 *rne*_WT_ | 0.0415 |
|  |  | *nalD*_T158P_ vs. *rne*_50bp_ | 0.8864 |
|  |  | *nalD*_T158P_ vs. *rne*::tn | 0.0241 |
|  |  | *nalD*_T158P_ vs. AzEvC10 *rne*_WT_ | >0.9999 |
|  |  | *rne*_50bp_ vs. *rne*::tn | >0.9999 |
|  |  | *rne*_50bp_ vs. AzEvC10 *rne*_WT_ | 0.2113 |
|  |  | *rne*::tn vs. AzEvC10 *rne*_WT_ | 0.0037 |
| Fig. 3C | Kruskal-Wallis test | WT vs. AzEvC10 | 0.0979 |
|  |  | WT vs. *nalD*_T158P_ | >0.9999 |
|  |  | WT vs. *rne*_50bp_ | >0.9999 |
|  |  | WT vs. *rne*::tn | 0.0062 |
|  |  | WT vs. AzEvC10 *rne*_WT_ | >0.9999 |
|  |  | AzEvC10 vs. *nalD*_T158P_ | 0.0011 |
|  |  | AzEvC10 vs. *rne*_50bp_ | >0.9999 |
|  |  | AzEvC10 vs. *rne*::tn | >0.9999 |
|  |  | AzEvC10 vs. AzEvC10 *rne*_WT_ | 0.0277 |
|  |  | *nalD*_T158P_ vs. *rne*_50bp_ | 0.4833 |
|  |  | *nalD*_T158P_ vs. *rne*::tn | <0.0001 |
|  |  | *nalD*_T158P_ vs. AzEvC10 *rne*_WT_ | >0.9999 |
|  |  | *rne*_50bp_ vs. *rne*::tn | 0.1891 |
|  |  | *rne*_50bp_ vs. AzEvC10 *rne*_WT_ | >0.9999 |
|  |  | *rne*::tn vs. AzEvC10 *rne*_WT_ | 0.0019 |
| Fig. 3D | Kruskal-Wallis test | WT vs. AzEvC10 | 0.0015 |
|  |  | WT vs. *nalD*_T158P_ | >0.9999 |
|  |  | WT vs. *rne*_50bp_ | 0.2070 |
|  |  | WT vs. *rne*::tn | <0.0001 |
|  |  | WT vs. AzEvC10 *rne*_WT_ | >0.9999 |
|  |  | AzEvC10 vs. *nalD*_T158P_ | 0.0020 |
|  |  | AzEvC10 vs. *rne*_50bp_ | >0.9999 |
|  |  | AzEvC10 vs. *rne*::tn | >0.9999 |
|  |  | AzEvC10 vs. AzEvC10 *rne*_WT_ | 0.5189 |
|  |  | *nalD*_T158P_ vs. *rne*_50bp_ | 0.1446 |
|  |  | *nalD*_T158P_ vs. *rne*::tn | <0.0001 |
|  |  | *nalD*_T158P_ vs. AzEvC10 *rne*_WT_ | >0.9999 |
|  |  | *rne*_50bp_ vs. *rne*::tn | 0.7213 |
|  |  | *rne*_50bp_ vs. AzEvC10 *rne*_WT_ | >0.9999 |
|  |  | *rne*::tn vs. AzEvC10 *rne*_WT_ | 0.0454 |
| Fig. 3E | Kruskal-Wallis test | WT vs. AzEvC10 | 0.0011 |
|  |  | WT vs. *nalD*_T158P_ | >0.9999 |
|  |  | WT vs. *rne*_50bp_ | 0.2010 |
|  |  | WT vs. *rne*::tn | 0.0031 |
|  |  | WT vs. AzEvC10 *rne*_WT_ | >0.9999 |
|  |  | AzEvC10 vs. *nalD*_T158P_ | <0.0001 |
|  |  | AzEvC10 vs. *rne*_50bp_ | >0.9999 |
|  |  | AzEvC10 vs. *rne*::tn | >0.9999 |
|  |  | AzEvC10 vs. AzEvC10 *rne*_WT_ | 0.3366 |
|  |  | *nalD*_T158P_ vs. *rne*_50bp_ | 0.0125 |
|  |  | *nalD*_T158P_ vs. *rne*::tn | 0.0001 |
|  |  | *nalD*_T158P_ vs. AzEvC10 *rne*_WT_ | 0.3583 |
|  |  | *rne*_50bp_ vs. *rne*::tn | >0.9999 |
|  |  | *rne*_50bp_ vs. AzEvC10 *rne*_WT_ | >0.9999 |
|  |  | *rne*::tn vs. AzEvC10 *rne*_WT_ | 0.4296 |
| Fig. 3F | Kruskal-Wallis test | WT vs. AzEvC10 | 0.0011 |
|  |  | WT vs. *nalD*_T158P_ | >0.9999 |
|  |  | WT vs. *rne*_50bp_ | 0.3177 |
|  |  | WT vs. *rne*::tn | 0.0004 |
|  |  | WT vs. AzEvC10 *rne*_WT_ | >0.9999 |
|  |  | AzEvC10 vs. *nalD*_T158P_ | 0.0005 |
|  |  | AzEvC10 vs. *rne*_50bp_ | >0.9999 |
|  |  | AzEvC10 vs. *rne*::tn | >0.9999 |
|  |  | AzEvC10 vs. AzEvC10 *rne*_WT_ | 0.1913 |
|  |  | *nalD*_T158P_ vs. *rne*_50bp_ | 0.1096 |
|  |  | *nalD*_T158P_ vs. *rne*::tn | 0.0002 |
|  |  | *nalD*_T158P_ vs. AzEvC10 *rne*_WT_ | >0.9999 |
|  |  | *rne*_50bp_ vs. *rne*::tn | >0.9999 |
|  |  | *rne*_50bp_ vs. AzEvC10 *rne*_WT_ | >0.9999 |
|  |  | *rne*::tn vs. AzEvC10 *rne*_WT_ | 0.0712 |
| Fig. 5A | ANOVA (One-way) | WT 3hpi vs. AzEvC10 3hpi | 0.9382 |
|  |  | WT 3hpi vs. WT 6hpi | 0.0013 |
|  |  | WT 3hpi vs. AzEvC10 6hpi | <0.0001 |
|  |  | AzEvC10 3hpi vs. WT 6hpi | 0.0026 |
|  |  | AzEvC10 3hpi vs. AzEvC10 6hpi | <0.0001 |
|  |  | WT 6hpi vs. AzEvC10 6hpi | 0.0084 |
| Fig. 5B | ANOVA (One-way) | WT 3hpi vs. AzEvC10 3hpi | 0.4904 |
|  |  | WT 3hpi vs. WT 6hpi | >0.9999 |
|  |  | WT 3hpi vs. AzEvC10 6hpi | 0.0010 |
|  |  | AzEvC10 3hpi vs. WT 6hpi | 0.3996 |
|  |  | AzEvC10 3hpi vs. AzEvC10 6hpi | 0.0163 |
|  |  | WT 6hpi vs. AzEvC10 6hpi | 0.0003 |
| Fig. 5C | ANOVA (One-way) | WT 3hpi vs. AzEvC10 3hpi | 0.0081 |
|  |  | WT 3hpi vs. WT 6hpi | 0.0085 |
|  |  | WT 3hpi vs. AzEvC10 6hpi | <0.0001 |
|  |  | AzEvC10 3hpi vs. WT 6hpi | >0.9999 |
|  |  | AzEvC10 3hpi vs. AzEvC10 6hpi | 0.0001 |
|  |  | WT 6hpi vs. AzEvC10 6hpi | <0.0001 |
| Fig. 5E | ANOVA (One-way) | Untreated vs. Fe(III) _10μM_ | <0.0001 |
|  |  | Untreated vs. Pch _10μM_ | <0.0001 |
|  |  | Untreated vs. Pch _Fe(III) 10μM_ | <0.0001 |
|  |  | Untreated vs. Pvd _10μM_ | 0.8619 |
|  |  | Untreated vs. Pvd _Fe(III) 10μM_ | 0.9995 |
|  |  | Fe(III) _10μM_ vs. Pch _10μM_ | <0.0001 |
|  |  | Fe(III) _10μM_ vs. Pch _Fe(III) 10μM_ | <0.0001 |
|  |  | Fe(III) _10μM_ vs. Pvd _10μM_ | <0.0001 |
|  |  | Fe(III) _10μM_ vs. Pvd _Fe(III) 10μM_ | <0.0001 |
|  |  | Pch _10μM_ vs. Pch _Fe(III) 10μM_ | <0.0001 |
|  |  | Pch _10μM_ vs. Pvd _10μM_ | <0.0001 |
|  |  | Pch _10μM_ vs. Pvd _Fe(III) 10μM_ | <0.0001 |
|  |  | Pch _Fe(III) 10μM_ vs. Pvd _10μM_ | <0.0001 |
|  |  | Pch _Fe(III) 10μM_ vs. Pvd _Fe(III) 10μM_ | <0.0001 |
|  |  | Pvd _10μM_ vs. Pvd _Fe(III) 10μM_ | 0.9595 |
| Fig. 5G | ANOVA (One-way) | Untreated vs. Fe(III) _10μM_ | <0.0001 |
|  |  | Untreated vs. Pch _10μM_ | <0.0001 |
|  |  | Untreated vs. Pch _Fe(III) 10μM_ | <0.0001 |
|  |  | Untreated vs. Pvd _10μM_ | 0.0098 |
|  |  | Untreated vs. Pvd _Fe(III) 10μM_ | 0.0720 |
|  |  | Fe(III) _10μM_ vs. Pch _10μM_ | <0.0001 |
|  |  | Fe(III) _10μM_ vs. Pch _Fe(III) 10μM_ | 0.0007 |
|  |  | Fe(III) _10μM_ vs. Pvd _10μM_ | <0.0001 |
|  |  | Fe(III) _10μM_ vs. Pvd _Fe(III) 10μM_ | <0.0001 |
|  |  | Pch _10μM_ vs. Pch _Fe(III) 10μM_ | <0.0001 |
|  |  | Pch _10μM_ vs. Pvd _10μM_ | <0.0001 |
|  |  | Pch _10μM_ vs. Pvd _Fe(III) 10μM_ | <0.0001 |
|  |  | Pch _Fe(III) 10μM_ vs. Pvd _10μM_ | <0.0001 |
|  |  | Pch _Fe(III) 10μM_ vs. Pvd _Fe(III) 10μM_ | <0.0001 |
|  |  | Pvd _10μM_ vs. Pvd _Fe(III) 10μM_ | 0.8341 |
| Fig. 6B | ANOVA (Two-way) | WT 0 vs. 100 | 0.0004 |
|  |  | WT 0 vs. 200 | 0.0001 |
|  |  | WT 0 vs. 375 | 0.0001 |
|  |  | WT 0 vs. 500 | 0.0001 |
|  |  | WT 0 vs. 750 | 0.0001 |
|  |  | AzEvC10 0 vs. 100 | <0.0001 |
|  |  | AzEvC10 0 vs. 200 | <0.0001 |
|  |  | AzEvC10 0 vs. 375 | <0.0001 |
|  |  | AzEvC10 0 vs. 500 | <0.0001 |
|  |  | AzEvC10 0 vs. 750 | <0.0001 |
|  |  | WT 0 vs. AzEvC10 0 | 0.0287 |
|  |  | WT 100 vs. AzEvC10 100 | 0.0013 |
|  |  | WT 200 vs. AzEvC10 200 | 0.0049 |
|  |  | WT 375 vs. AzEvC10 375 | 0.0587 |
|  |  | WT 500 vs. AzEvC10 500 | 0.1668 |
|  |  | WT 750 vs. AzEvC10 750 | 0.0722 |
| Fig. 6C  Bacterial burden | ANOVA (One-way) | WT vs. WT+ gallium | >0.9999 |
|  |  | WT vs. AzEvC10 | <0.0001 |
|  |  | WT vs. AzEvC10+ gallium | 0.0002 |
|  |  | WT+ gallium vs. AzEvC10 | <0.0001 |
|  |  | WT+ gallium vs. AzEvC10+ gallium | 0.0002 |
|  |  | AzEvC10 vs. AzEvC10+ gallium | 0.1278 |
| Fig. 6C  Pyochelin | Kruskal-Wallis test | WT vs. WT+ gallium | >0.9999 |
|  |  | WT vs. AzEvC10 | 0.2026 |
|  |  | WT vs. AzEvC10+ gallium | >0.9999 |
|  |  | WT+ gallium vs. AzEvC10 | 0.0374 |
|  |  | WT+ gallium vs. AzEvC10+ gallium | >0.9999 |
|  |  | AzEvC10 vs. AzEvC10+ gallium | 0.0100 |
| Fig. 6C  Pyoverdine | Kruskal-Wallis test | WT vs. WT+ gallium | >0.9999 |
|  |  | WT vs. AzEvC10 | 0.0017 |
|  |  | WT vs. AzEvC10+ gallium | 0.1829 |
|  |  | WT+ gallium vs. AzEvC10 | 0.0012 |
|  |  | WT+ gallium vs. AzEvC10+ gallium | 0.1485 |
|  |  | AzEvC10 vs. AzEvC10+ gallium | 0.8499 |
| Fig. 6D  Bacterial burden | ANOVA (One-way) | WT vs. WT+ gallium | 0.0131 |
|  |  | WT vs. AzEvC10 | <0.0001 |
|  |  | WT vs. AzEvC10+ gallium | 0.1031 |
|  |  | WT+ gallium vs. AzEvC10 | <0.0001 |
|  |  | WT+ gallium vs. AzEvC10+ gallium | <0.0001 |
|  |  | AzEvC10 vs. AzEvC10+ gallium | 0.0005 |
| Fig. 6D Pyochelin | Kruskal-Wallis test | WT vs. WT+ gallium | >0.9999 |
|  |  | WT vs. AzEvC10 | 0.0087 |
|  |  | WT vs. AzEvC10+ gallium | 0.5185 |
|  |  | WT+ gallium vs. AzEvC10 | 0.0002 |
|  |  | WT+ gallium vs. AzEvC10+ gallium | 0.0423 |
|  |  | AzEvC10 vs. AzEvC10+ gallium | 0.8499 |
| Fig. 6D Pyoverdine | Kruskal-Wallis test | WT vs. WT+ gallium | >0.9999 |
|  |  | WT vs. AzEvC10 | 0.0151 |
|  |  | WT vs. AzEvC10+ gallium | 0.7249 |
|  |  | WT+ gallium vs. AzEvC10 | <0.0001 |
|  |  | WT+ gallium vs. AzEvC10+ gallium | 0.0256 |
|  |  | AzEvC10 vs. AzEvC10+ gallium | 0.8499 |
| Fig. 6E  Bacterial burden | ANOVA (One-way) | WT vs. WT+ gallium | <0.0001 |
|  |  | WT vs. AzEvC10 | 0.9671 |
|  |  | WT vs. AzEvC10+ gallium | <0.0001 |
|  |  | WT+ gallium vs. AzEvC10 | <0.0001 |
|  |  | WT+ gallium vs. AzEvC10+ gallium | 0.0008 |
|  |  | AzEvC10 vs. AzEvC10+ gallium | <0.0001 |
| Fig. 6E Pyochelin | Kruskal-Wallis test | WT vs. WT+ gallium | 0.0449 |
|  |  | WT vs. AzEvC10 | 0.4960 |
|  |  | WT vs. AzEvC10+ gallium | >0.9999 |
|  |  | WT+ gallium vs. AzEvC10 | <0.0001 |
|  |  | WT+ gallium vs. AzEvC10+ gallium | 0.4960 |
|  |  | AzEvC10 vs. AzEvC10+ gallium | 0.0449 |
| Fig. 6E Pyoverdine | Kruskal-Wallis test | WT vs. WT+ gallium | 0.0197 |
|  |  | WT vs. AzEvC10 | 0.8499 |
|  |  | WT vs. AzEvC10+ gallium | 0.8499 |
|  |  | WT+ gallium vs. AzEvC10 | <0.0001 |
|  |  | WT+ gallium vs. AzEvC10+ gallium | 0.8499 |
|  |  | AzEvC10 vs. AzEvC10+ gallium | 0.0197 |
| Fig. 7F | Fisher’s exact test (Two-sided) | N- (catalytic) vs. C- (degradosome) | <0.0001 |
| Fig S1A  Neutrophils | T-test (unpaired, two-tailed) | WT vs. AzEvC10 | 0.8002 |
| Fig. S1A  AEC | T-test (unpaired, two-tailed) | WT vs. AzEvC10 | 0.7035 |
| Fig. S1C | T-test (unpaired, two-tailed) | WT vs. AzEvC10 | <0.0001 |
| Fig. S1D | T-test (unpaired, two-tailed) | WT vs. AzEvC10 | <0.0001 |
| Fig. S2B | ANOVA (One-way) | WT vs. AzEvC10 | 0.3730 |
|  |  | WT vs. AzEvC10::*nalD* | <0.0001 |
|  |  | AzEvC10 vs. AzEvC10::*nalD* | <0.0001 |
| Fig. S2C | Kruskal-Wallis test | WT vs. AzEvC10 | 0.1915 |
|  |  | WT vs. AzEvC10::*nalD* | 0.8114 |
|  |  | AzEvC10 vs. AzEvC10::*nalD* | 0.0094 |
| Fig. S2D | ANOVA (One-way) | WT vs. AzEvC10 | 0.0081 |
|  |  | WT vs. AzEvC10::*nalD* | 0.0333 |
|  |  | AzEvC10 vs. AzEvC10::*nalD* | 0.6388 |
| Fig. S2E | ANOVA (One-way) | WT vs. AzEvC10 | 0.0030 |
|  |  | WT vs. AzEvC10::*nalD* | 0.0014 |
|  |  | AzEvC10 vs. AzEvC10::*nalD* | 0.6022 |
| Fig. S2G | ANOVA (One-way) | WT vs. Δ*mexAB* | <0.0001 |
|  |  | WT vs. AzEvC10 | <0.0001 |
|  |  | WT vs. AzEvC10 Δ*mexAB* | <0.0001 |
|  |  | Δ*mexAB* vs. AzEvC10 | <0.0001 |
|  |  | Δ*mexAB* vs. AzEvC10 Δ*mexAB* | 0.7771 |
|  |  | AzEvC10 vs. AzEvC10 Δ*mexAB* | <0.0001 |
| Fig. S2H | ANOVA (One-way) | WT vs. Δ*mexAB* | >0.9999 |
|  |  | WT vs. AzEvC10 | 0.0257 |
|  |  | WT vs. AzEvC10 Δ*mexAB* | 0.0125 |
|  |  | Δ*mexAB* vs. AzEvC10 | 0.0258 |
|  |  | Δ*mexAB* vs. AzEvC10 Δ*mexAB* | 0.0125 |
|  |  | AzEvC10 vs. AzEvC10 Δ*mexAB* | 0.9478 |
| Fig. S2I | ANOVA (One-way) | WT vs. Δ*mexAB* | 0.9857 |
|  |  | WT vs. AzEvC10 | 0.0009 |
|  |  | WT vs. AzEvC10 Δ*mexAB* | 0.0024 |
|  |  | Δ*mexAB* vs. AzEvC10 | 0.0013 |
|  |  | Δ*mexAB* vs. AzEvC10 Δ*mexAB* | 0.0035 |
|  |  | AzEvC10 vs. AzEvC10 Δ*mexAB* | 0.8059 |
| Fig. S3F  Pyochelin | Mann-Whitney test | WT vs. AzEvC10 | 0.0519 |
| Fig. S3F  Pyoverdine | T-test (unpaired, two-tailed) | WT vs. AzEvC10 | 0.2763 |
| Fig. S4A  Annexin MFI | ANOVA (One-way) | WT 3hpi vs. AzEvC10 3hpi | <0.0001 |
|  |  | WT 3hpi vs. WT 6hpi | 0.3016 |
|  |  | WT 3hpi vs. AzEvC10 6hpi | 0.4317 |
|  |  | AzEvC10 3hpi vs. WT 6hpi | 0.0013 |
|  |  | AzEvC10 3hpi vs. AzEvC10 6hpi | 0.9900 |
|  |  | WT 6hpi vs. AzEvC10 6hpi | 0.0129 |
| Fig. S4A  PI MFI | ANOVA (One-way) | WT 3hpi vs. AzEvC10 3hpi | 0.0087 |
|  |  | WT 3hpi vs. WT 6hpi | 0.0067 |
|  |  | WT 3hpi vs. AzEvC10 6hpi | 0.0004 |
|  |  | AzEvC10 3hpi vs. WT 6hpi | <0.0001 |
|  |  | AzEvC10 3hpi vs. AzEvC10 6hpi | 0.1427 |
|  |  | WT 6hpi vs. AzEvC10 6hpi | 0.0011 |
|  |  | WT 6hpi vs. AzEvC10 6hpi | 0.0208 |
| Fig. S4B | T-test (unpaired, two-tailed) | WT vs. AzEvC10 | 0.9414 |
| Fig S4C | ANOVA (One-way) | Fe(III) _100μM_ vs. Pch _100μM_ | >0.9999 |
|  |  | Fe(III) _100μM_ vs. Pch _Fe(III) 100μM_ | <0.0001 |
|  |  | Fe(III) _100μM_ vs. Pvd _100μM_ | 0.9999 |
|  |  | Fe(III) _100μM_ vs. Pvd _Fe(III) 100μM_ | 0.9997 |
|  |  | Pch _100μM_ vs. Pch _Fe(III) 100μM_ | <0.0001 |
|  |  | Pch _100μM_ vs. Pvd _100μM_ | >0.9999 |
|  |  | Pch _100μM_ vs. Pvd _Fe(III) 100μM_ | 0.9999 |
|  |  | Pch _Fe(III) 100μM_ vs. Pvd _100μM_ | <0.0001 |
|  |  | Pch _Fe(III) 100μM_ vs. Pvd _Fe(III) 100μM_ | <0.0001 |
|  |  | Pvd _100μM_ vs. Pvd _Fe(III) 100μM_ | >0.9999 |
| Fig S4D | ANOVA (One-way) | Fe(III) _100μM_ vs. Pch _100μM_ | >0.9999 |
|  |  | Fe(III) _100μM_ vs. Pch _Fe(III) 100μM_ | <0.0001 |
|  |  | Fe(III) _100μM_ vs. Pvd _100μM_ | 0.9999 |
|  |  | Fe(III) _100μM_ vs. Pvd _Fe(III) 100μM_ | >0.9999 |
|  |  | Pch _100μM_ vs. Pch _Fe(III) 100μM_ | <0.0001 |
|  |  | Pch _100μM_ vs. Pvd _100μM_ | >0.9999 |
|  |  | Pch _100μM_ vs. Pvd _Fe(III) 100μM_ | >0.9999 |
|  |  | Pch _Fe(III) 100μM_ vs. Pvd _100μM_ | <0.0001 |
|  |  | Pch _Fe(III) 100μM_ vs. Pvd _Fe(III) 100μM_ | <0.0001 |
|  |  | Pvd _100μM_ vs. Pvd _Fe(III) 100μM_ | >0.9999 |
| Fig. S5A | Kruskal-Wallis test | 0 vs. 500 | 0.2617 |
|  |  | 0 vs. 750 | 0.7267 |
|  |  | 0 vs. 1000 | 0.6134 |
|  |  | 0 vs. 2000 | 0.0110 |
| Fig. S5C | ANOVA (One-way) | WT vs. WT+gallium | 0.9465 |
|  |  | WT vs. AzEvC10 | 0.7885 |
|  |  | WT vs. AzEvC10+gallium | 0.8486 |
|  |  | WT+gallium vs. AzEvC10 | 0.4934 |
|  |  | WT+gallium vs. AzEvC10+gallium | 0.9927 |
|  |  | AzEvC10 vs. AzEvC10+gallium | 0.3641 |
| Fig. S5D | ANOVA (One-way) | WT vs. WT+gallium | <0.0001 |
|  |  | WT vs. AzEvC10 | 0.0188 |
|  |  | WT vs. AzEvC10+gallium | <0.0001 |
|  |  | WT+gallium vs. AzEvC10 | 0.0048 |
|  |  | WT+gallium vs. AzEvC10+gallium | <0.0001 |
|  |  | AzEvC10 vs. AzEvC10+gallium | <0.0001 |
| Fig. S5E | ANOVA (One-way) | WT vs. WT+gallium | <0.0001 |
|  |  | WT vs. AzEvC10 | 0.0116 |
|  |  | WT vs. AzEvC10+gallium | <0.0001 |
|  |  | WT+gallium vs. AzEvC10 | <0.0001 |
|  |  | WT+gallium vs. AzEvC10+gallium | 0.7187 |
|  |  | AzEvC10 vs. AzEvC10+gallium | <0.0001 |
| Fig. S5F | ANOVA (One-way) | WT vs. WT+gallium | 0.5880 |
|  |  | WT vs. AzEvC10 | <0.0001 |
|  |  | WT vs. AzEvC10+gallium | 0.0628 |
|  |  | WT+gallium vs. AzEvC10 | <0.0001 |
|  |  | WT+gallium vs. AzEvC10+gallium | 0.0106 |
|  |  | AzEvC10 vs. AzEvC10+gallium | <0.0001 |
| Fig. S5G | ANOVA (One-way) | WT vs. WT+gallium | <0.0001 |
|  |  | WT vs. AzEvC10 | 0.0006 |
|  |  | WT vs. AzEvC10+gallium | <0.0001 |
|  |  | WT+gallium vs. AzEvC10 | <0.0001 |
|  |  | WT+gallium vs. AzEvC10+gallium | >0.9999 |
|  |  | AzEvC10 vs. AzEvC10+gallium | <0.0001 |
| Fig. S5H | ANOVA (One-way) | WT vs. WT+gallium | 0.0004 |
|  |  | WT vs. AzEvC10 | 0.0324 |
|  |  | WT vs. AzEvC10+gallium | 0.0013 |
|  |  | WT+gallium vs. AzEvC10 | 0.0204 |
|  |  | WT+gallium vs. AzEvC10+gallium | 0.5986 |
|  |  | AzEvC10 vs. AzEvC10+gallium | 0.1224 |
| Fig. S5I | ANOVA (One-way) | Ga(III) _10μM_ vs. Ga(III) _100μM_ | >0.9999 |
|  |  | Ga(III) _10μM_ vs. Pch _10μM_ | >0.9999 |
|  |  | Ga(III) _10μM_ vs. Pch _Fe(III) 10μM_ | 0.7987 |
|  |  | Ga(III) _10μM_ vs. Pch _Ga(III) 10μM_ | 0.9938 |
|  |  | Ga(III) _10μM_ vs. Pch _100μM_ | >0.9999 |
|  |  | Ga(III) _10μM_ vs. Pch _Fe(III) 100μM_ | <0.0001 |
|  |  | Ga(III) _10μM_ vs. Pch _Ga(III) 100μM_ | 0.6141 |
|  |  | Ga(III) _100μM_ vs. Pch _10μM_ | >0.9999 |
|  |  | Ga(III) _100μM_ vs. Pch _Fe(III) 10μM_ | 0.6788 |
|  |  | Ga(III) _100μM_ vs. Pch _Ga(III) 10μM_ | 0.9734 |
|  |  | Ga(III) _100μM_ vs. Pch _100μM_ | >0.9999 |
|  |  | Ga(III) _100μM_ vs. Pch _Fe(III) 100μM_ | <0.0001 |
|  |  | Ga(III) _100μM_ vs. Pch _Ga(III) 100μM_ | 0.4859 |
|  |  | Pch _10μM_ vs. Pch _Fe(III) 10μM_ | 0.5783 |
|  |  | Pch _10μM_ vs. Pch _Ga(III) 10μM_ | 0.9391 |
|  |  | Pch _10μM_ vs. Pch _100μM_ | >0.9999 |
|  |  | Pch _10μM_ vs. Pch _Fe(III) 100μM_ | <0.0001 |
|  |  | Pch _10μM_ vs. Pch _Ga(III) 100μM_ | 0.3935 |
|  |  | Pch _Fe(III) 10μM_ vs. Pch _Ga(III) 10μM_ | 0.9936 |
|  |  | Pch _Fe(III) 10μM_ vs. Pch _100μM_ | 0.7642 |
|  |  | Pch _Fe(III) 10μM_ vs. Pch _Fe(III) 100μM_ | <0.0001 |
|  |  | Pch _Fe(III) 10μM_ vs. Pch _Ga(III) 100μM_ | >0.9999 |
|  |  | Pch _Ga(III) 10μM_ vs. Pch _100μM_ | 0.9898 |
|  |  | Pch _Ga(III) 10μM_ vs. Pch _Fe(III) 100μM_ | <0.0001 |
|  |  | Pch _Ga(III) 10μM_ vs. Pch _Ga(III) 100μM_ | 0.9525 |
|  |  | Pch _100μM_ vs. Pch _Fe(III) 100μM_ | <0.0001 |
|  |  | Pch _100μM_ vs. Pch _Ga(III) 100μM_ | 0.5747 |
|  |  | Pch _Fe(III) 100μM_ vs. Pch _Ga(III) 100μM_ | <0.0001 |
| Fig. S5K | ANOVA (One-way) | Ga(III) _10μM_ vs. Ga(III) _100μM_ | >0.9999 |
|  |  | Ga(III) _10μM_ vs. Pch _10μM_ | >0.9999 |
|  |  | Ga(III) _10μM_ vs. Pch _Fe(III) 10μM_ | >0.9999 |
|  |  | Ga(III) _10μM_ vs. Pch _Ga(III) 10μM_ | >0.9999 |
|  |  | Ga(III) _10μM_ vs. Pch _100μM_ | >0.9999 |
|  |  | Ga(III) _10μM_ vs. Pch _Fe(III) 100μM_ | <0.0001 |
|  |  | Ga(III) _10μM_ vs. Pch _Ga(III) 100μM_ | 0.9984 |
|  |  | Ga(III) _100μM_ vs. Pch _10μM_ | >0.9999 |
|  |  | Ga(III) _100μM_ vs. Pch _Fe(III) 10μM_ | >0.9999 |
|  |  | Ga(III) _100μM_ vs. Pch _Ga(III) 10μM_ | >0.9999 |
|  |  | Ga(III) _100μM_ vs. Pch _100μM_ | >0.9999 |
|  |  | Ga(III) _100μM_ vs. Pch _Fe(III) 100μM_ | <0.0001 |
|  |  | Ga(III) _100μM_ vs. Pch _Ga(III) 100μM_ | 0.9956 |
|  |  | Pch _10μM_ vs. Pch _Fe(III) 10μM_ | >0.9999 |
|  |  | Pch _10μM_ vs. Pch _Ga(III) 10μM_ | >0.9999 |
|  |  | Pch _10μM_ vs. Pch _100μM_ | >0.9999 |
|  |  | Pch _10μM_ vs. Pch _Fe(III) 100μM_ | <0.0001 |
|  |  | Pch _10μM_ vs. Pch _Ga(III) 100μM_ | 0.9986 |
|  |  | Pch _Fe(III) 10μM_ vs. Pch _Ga(III) 10μM_ | >0.9999 |
|  |  | Pch _Fe(III) 10μM_ vs. Pch _100μM_ | >0.9999 |
|  |  | Pch _Fe(III) 10μM_ vs. Pch _Fe(III) 100μM_ | <0.0001 |
|  |  | Pch _Fe(III) 10μM_ vs. Pch _Ga(III) 100μM_ | 0.9996 |
|  |  | Pch _Ga(III) 10μM_ vs. Pch _100μM_ | >0.9999 |
|  |  | Pch _Ga(III) 10μM_ vs. Pch _Fe(III) 100μM_ | <0.0001 |
|  |  | Pch _Ga(III) 10μM_ vs. Pch _Ga(III) 100μM_ | 0.9984 |
|  |  | Pch _100μM_ vs. Pch _Fe(III) 100μM_ | <0.0001 |
|  |  | Pch _100μM_ vs. Pch _Ga(III) 100μM_ | 0.9971 |
|  |  | Pch _Fe(III) 100μM_ vs. Pch _Ga(III) 100μM_ | <0.0001 |
